# Supplementary material for: Regiodivergent biosynthesis of bridged bicyclononanes
Source: Nat Commun. 2024 May 28;15:4525. doi: 10.1038/s41467-024-48879-w (PMC11133429; doi:10.1038/s41467-024-48879-w)
Supplement: Supplementary file 1 — Supplementary information [file 41467_2024_48879_MOESM1_ESM.pdf]

SUPPLEMENTARY INFORMATION

Regiodivergent biosynthesis of bridged bicyclononanes

Lukas Ernst[1]*, Hui Lyu[2], Pi Liu[3], Christian Paetz[2], Hesham MB Sayed[1,4], Tomke Meents[1], Hongwu Ma[3], Ludger Beerhues[1,5], Islam El-Awaad[1,4,5]* & Benye Liu[1,5]*

[1]Technische Universität Braunschweig, Institute of Pharmaceutical Biology, Braunschweig 38106, Germany.

[2]Max Planck Institute for Chemical Ecology, NMR/Biosynthesis Group, Jena 07745, Germany.

[3]Chinese Academy of Sciences, Tianjin Institute of Industrial Biotechnology, Biodesign Center, Key Laboratory of Engineering Biology for Low-carbon Manufacturing, Tianjin 300308, China.

[4]Assiut University, Faculty of Pharmacy, Department of Pharmacognosy, Assiut 71526, Egypt.

[5]Technische Universität Braunschweig, Center of Pharmaceutical Engineering, Braunschweig 38106, Germany.

*Corresponding authors. Email: lukas.ernst@tu-braunschweig.de, islam.elawaad@tu-braunschweig.de, b.liu@tu-braunschweig.de

Table of Contents

Supplementary Figures **3**

Fig. 1 | Chemical shifts of isolated 7-*epi*-nemorosone **3** 3

Fig. 2 | Chemical shifts of isolated 7-*epi*-clusianone **5** 4

Fig. 3 | Chemical shifts of isolated nemosampsone **4** 5

Fig. 4 | Recorded ^{1}H spectrum of nemosampsone **4** 6

Fig. 5 | Recorded ^{13}C spectrum of nemosampsone **4** 6

Fig. 6 | Recorded ^{1}H–^{1}H COSY spectrum of nemosampsone **4** 7

Fig. 7 | Superimposed ^{1}H–^{13}C HSQC and ^{1}H–^{13}C HMBC spectra of nemosampsone **4** I 7

Fig. 8 | Superimposed ^{1}H–^{13}C HSQC and ^{1}H–^{13}C HMBC spectra of nemosampsone **4** II 8

Fig. 9 | Section of recorded ^{1}H–^{1}H ROESY spectrum of nemosampsone **4** I 8

Fig. 10 | Section of recorded ^{1}H–^{1}H ROESY spectrum of nemosampsone **4** II 9

Fig. 11 | Chemical shifts of isolated grandone **1** 9

Fig. 12 | Chemical shifts of isolated kolanone **2** 10

Fig. 13 | *Hs*CPTa- and *Hs*CPTb-catalysed reactions in the context of PPAP biosynthesis 10

Fig. 14 | Biosynthesis of prenylated xanthones in *H. sampsonii* 11

Fig. 15 | Phylogenetic estimation of putative PTs 11

Fig. 16 | Confirmation of product identities 12

Fig. 17 | Characterization of *Hs*CPTa and *Hs*CPTb 13

Fig. 18 | Localization of *Hs*CPTa and *Hs*CPTb in transiently transformed *N. benthamiana* leaves 14

Fig. 19 | Refinement of modelled *Hs*CPTa binding cavity 14

Fig. 20 | Donor substrate docking 15

Fig. 21 | Prenylative Cyclization: Stereochemistry and optical rotation 15

Fig. 22 | Analysis of *Hs*CPTb/*Hs*CPTa C-terminal exchange chimeras 16

Fig. 23 |Molecular modelling of HsCPTa/b chimeras and substrate docking 17

Fig. 24 | Summary of tested mutants 18

Fig. 25 |Examples of PPAP structures with aliphatic acyl groups from *H. perforatum* 19

Fig. 26 | Metabolite levels and gene expression 19

Fig. 27 | Chemical shifts of isolated 7-*epi*-secohyperforin **6** 20

Fig. 28 | Alignment of MS/MS spectra 21

Supplementary Tables **22**

Table 1 | ^{1}H and ^{13}C shifts, J_{HH} coupling constants and observed 2D-correlations of compound **4** 22

Table 2 | Empirical determination of C-7 stereochemistry by comparison of key chemical shifts 23

Table 3 | Comparison of chemical shifts reported for secohyperforin and extracted from **6** 24

Table 4 | FPKM values in various *H. perforatum* tissues for the *Hp*CPTa1-encoding transcripts 25

Table 5 | Primers used for plasmid construction, mutagenesis, and RT-qPCR 25

Other NMR spectra **27**

Supplementary References **37**

Supplementary Figures

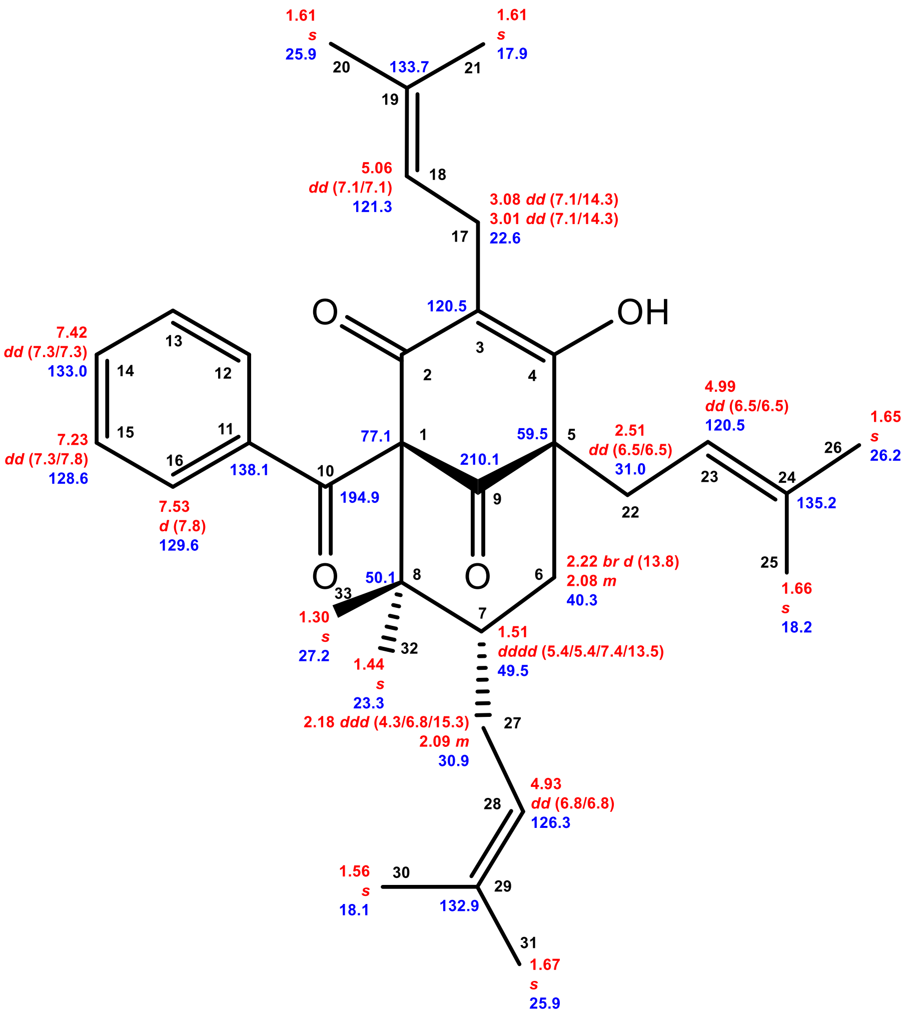

Supplementary Fig. 1 | Chemical shifts of isolated 7-*epi*-nemorosone **3**. Red: ^{1}H chemical shifts (δ ppm, *mult*., $^{3}J_{HH}$ in Hz). Blue: ^{13}C chemical shifts (δ ppm).

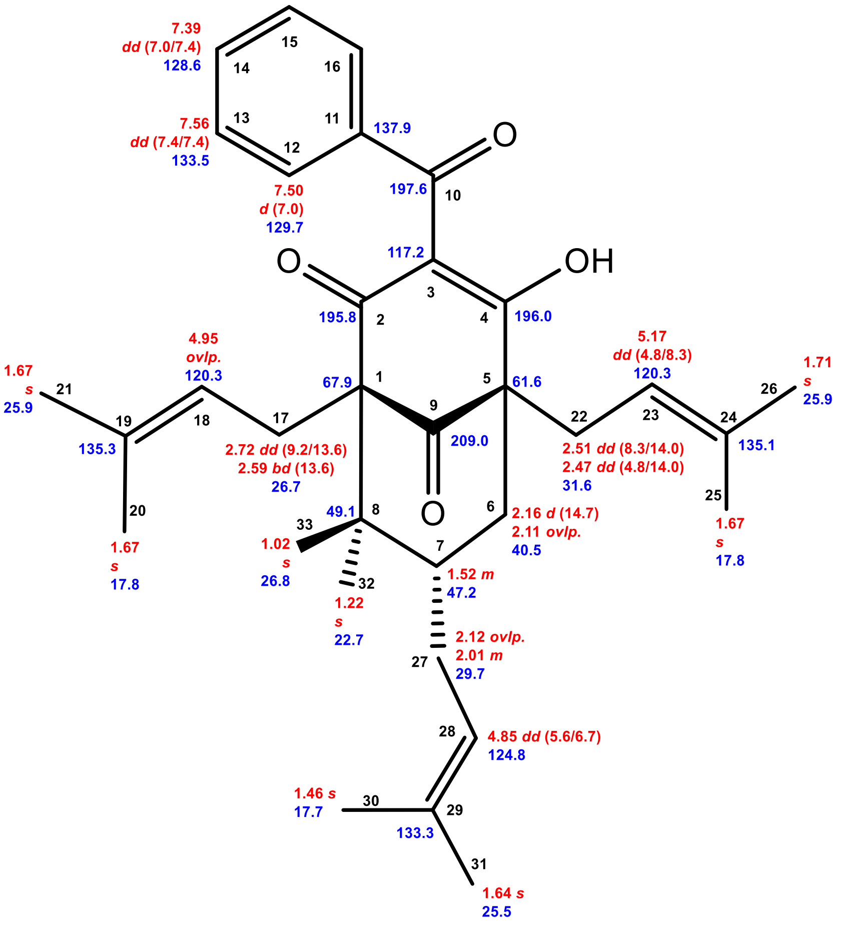

Supplementary Fig. 2 | Chemical shifts of isolated 7-*epi*-clusianone **5**. Red: ^{1}H chemical shifts (δ ppm, *mult.*, $^{3}J_{HH}$ in Hz). Blue: ^{13}C chemical shifts (δ ppm).

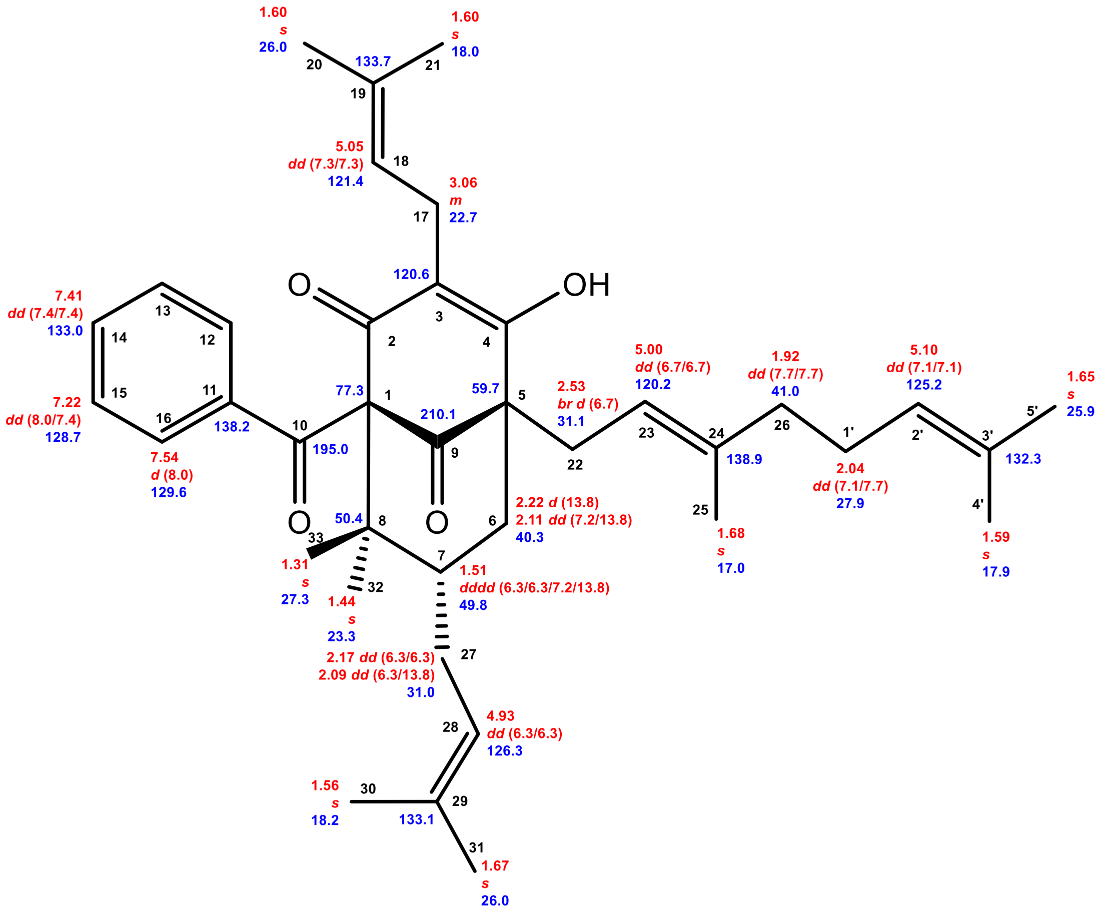

Supplementary Fig. 3 | Chemical shifts of nemosampsone **4**. Red: ^{1}H chemical shifts (δ ppm, *mult*., $^{3}J_{HH}$ in Hz). Blue: ^{13}C chemical shifts (δ ppm).

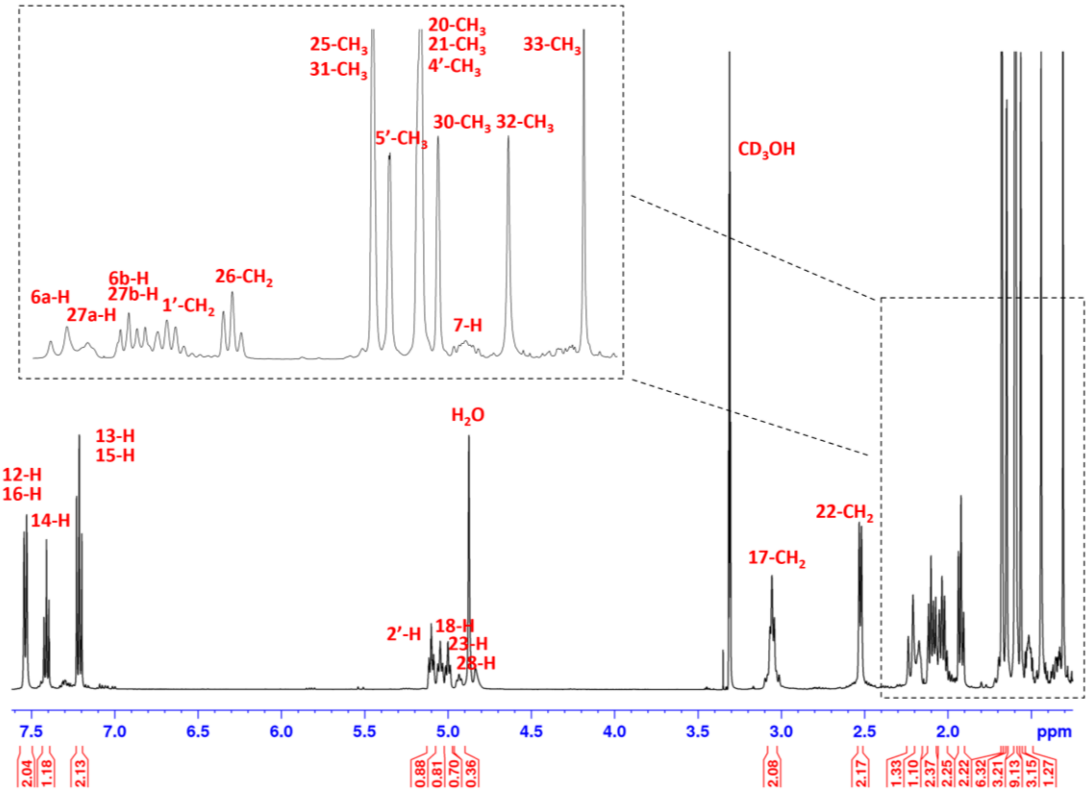

Supplementary Fig. 4 | Recorded ^{1}H spectrum of nemosampsone **4**, measured in CD_3OH at 500.13 MHz.

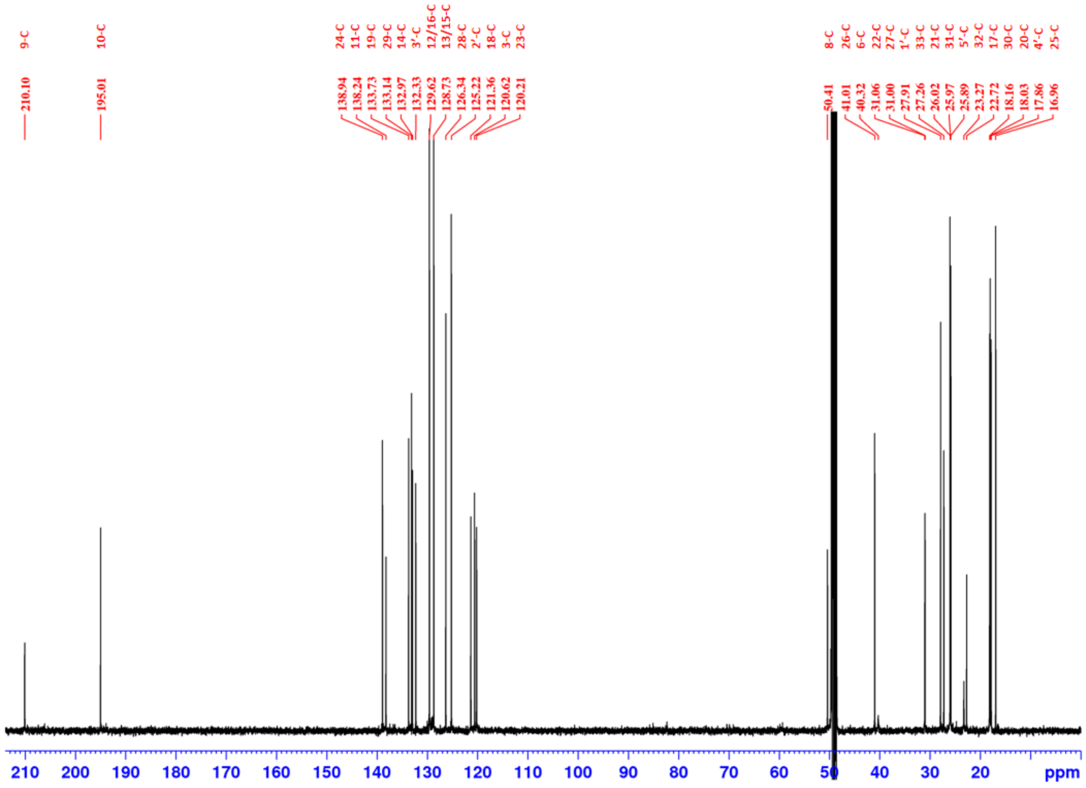

Supplementary Fig. 5 | Recorded ^{13}C spectrum of nemosampsone **4**, measured in CD_3OH at 125.75 MHz.

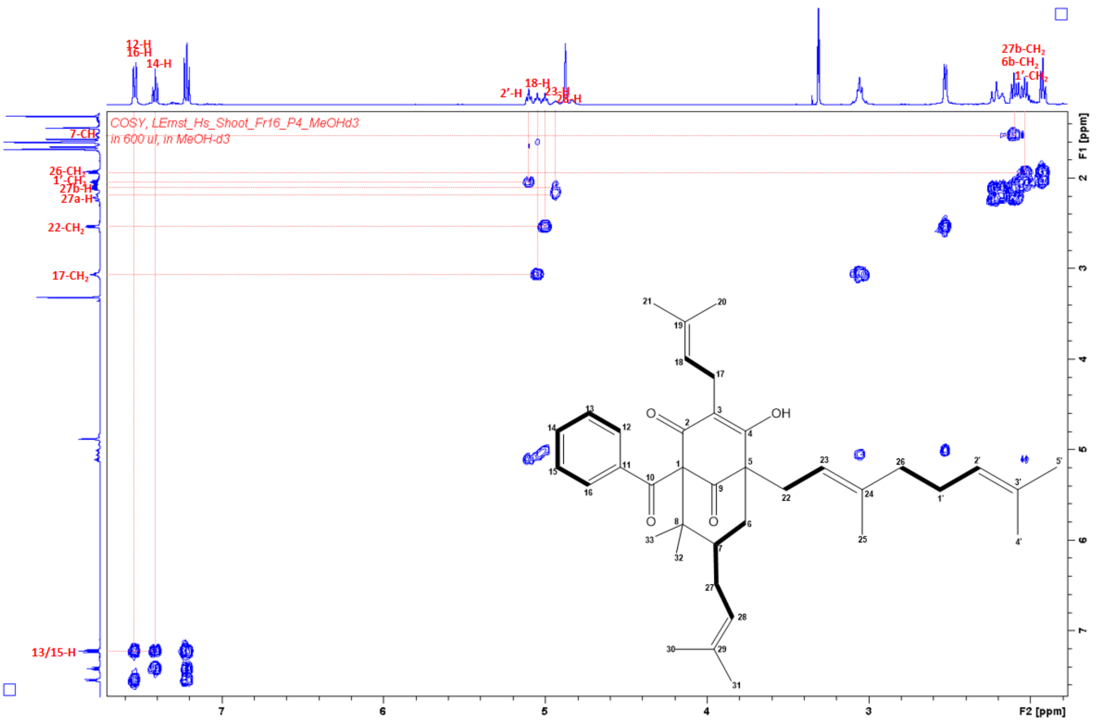

Supplementary Fig. 6 | Recorded $^{1}H–^{1}H$ COSY spectrum of nemosampsone **4**, measured in CD_3OH.

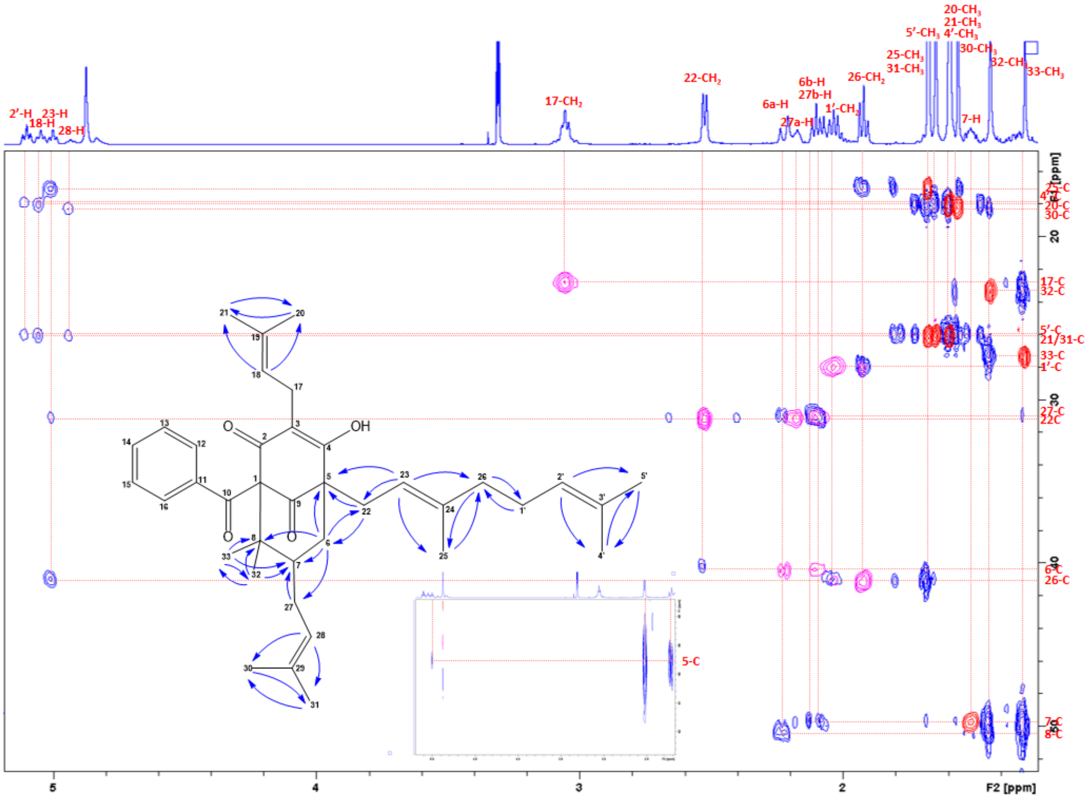

Supplementary Fig. 7 | Superimposed $^{1}H–^{13}C$ HSQC and $^{1}H–^{13}C$ HMBC spectra of nemosampsone **4**, measured in CD_3OH. Signals are shown in the range of δ_C 15–52 ppm. Correlations from H-6, H-22, and H-23 to C-5 required higher intensity and are shown as a cut-in.

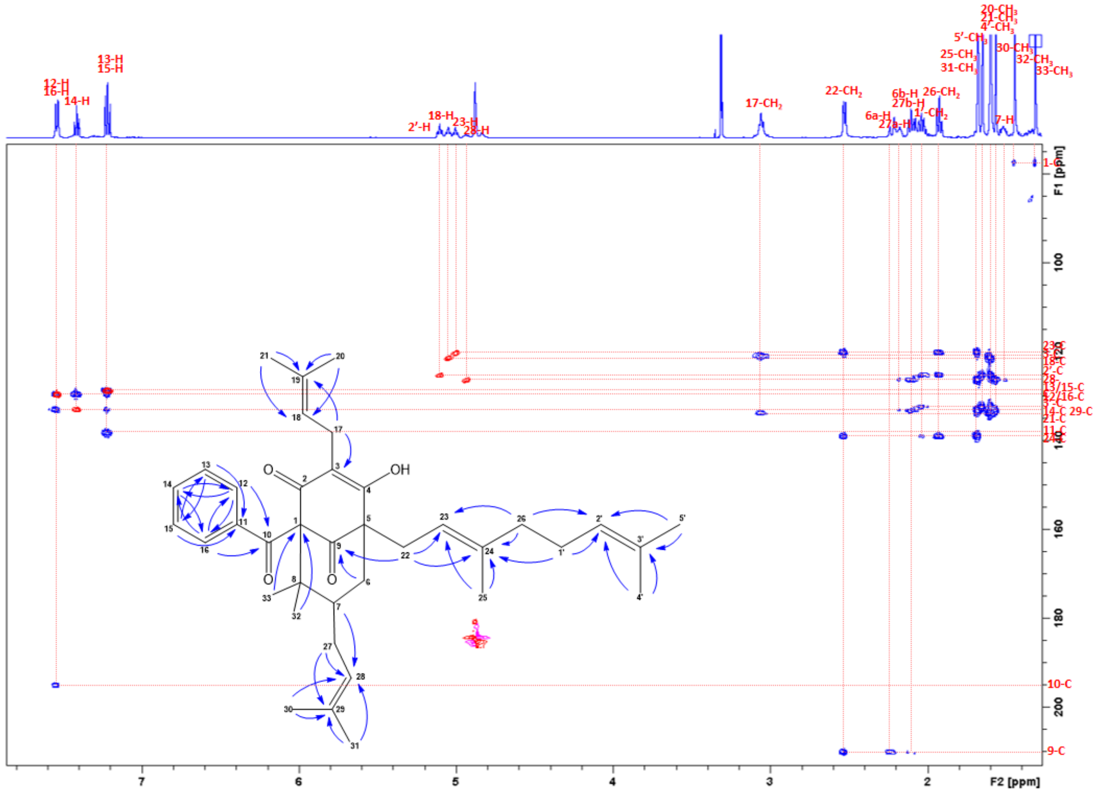

Supplementary Fig. 8 | Superimposed ^{1}H–^{13}C HSQC and ^{1}H–^{13}C HMBC spectra of nemosampsone **4**, measured in CD_3OH. Signals are shown in the range of δ_C 75–215 ppm.

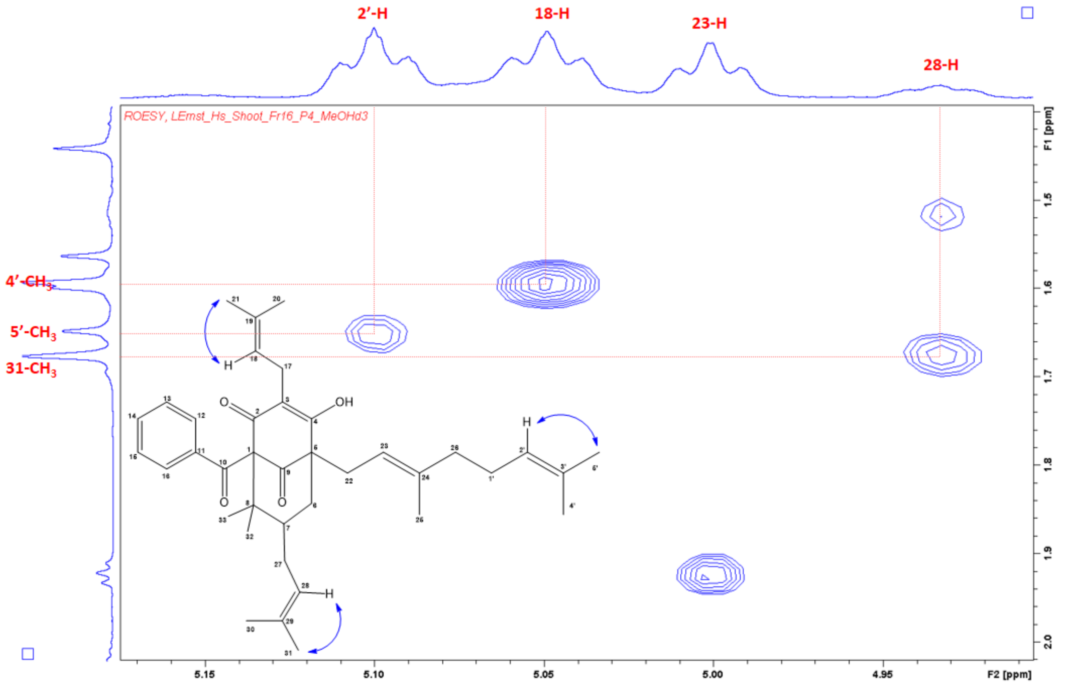

Supplementary Fig. 9 | Section of recorded ^{1}H–^{1}H ROESY spectrum of nemosampsone **4**, measured in CD_3OH. Signals are shown in the range of F1 δ_H 4.91–5.17 ppm and F2 δ_H 1.4–2.0 ppm.

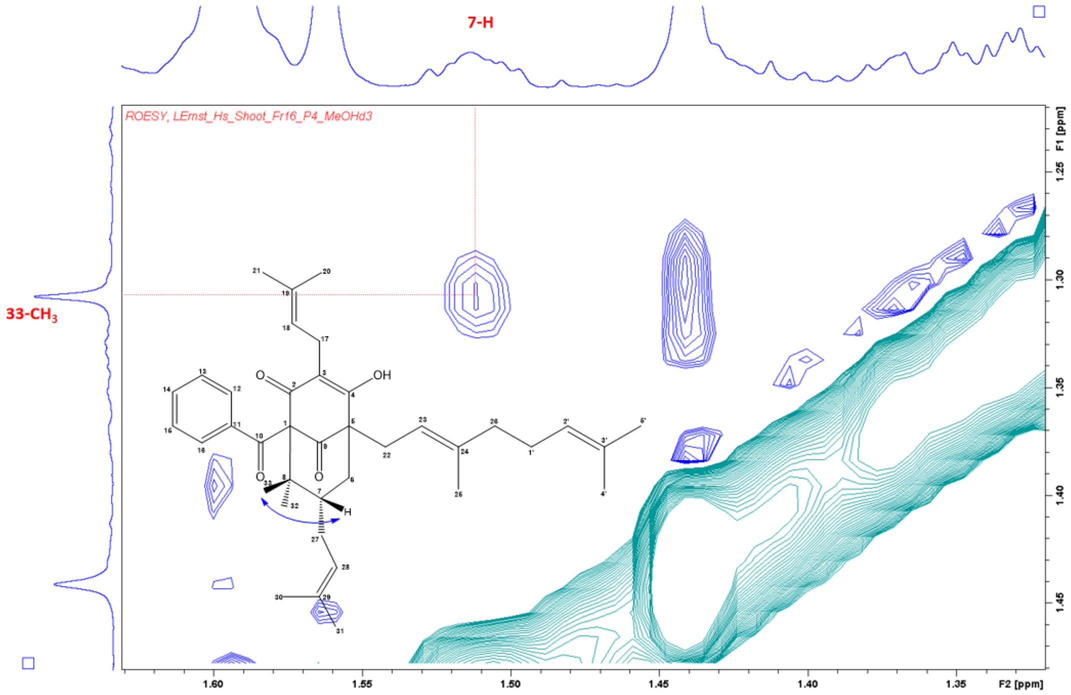

Supplementary Fig. 10 | Section of recorded ^{1}H–^{1}H ROESY spectrum of nemosampsone **4**, measured in CD_3OH. Signals are shown in the range of F1 δ_H 1.32–1.63 ppm and F2 δ_H 1.22–1.48 ppm.

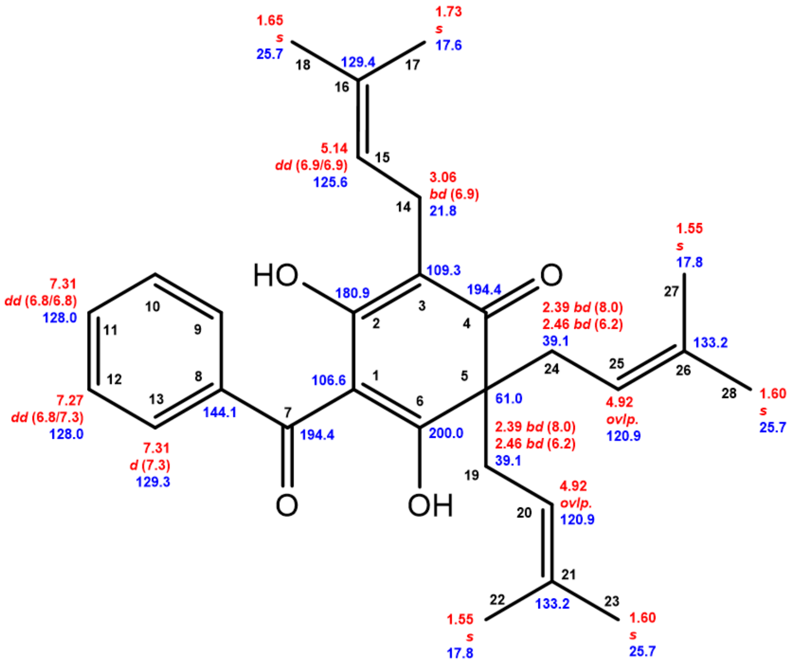

Supplementary Fig. 11 | Chemical shifts of isolated grandone **1**. Red: ^{1}H chemical shifts (δ ppm, *mult.*, $^{3}J_{HH}$ in Hz). Blue: ^{13}C chemical shifts (δ ppm).

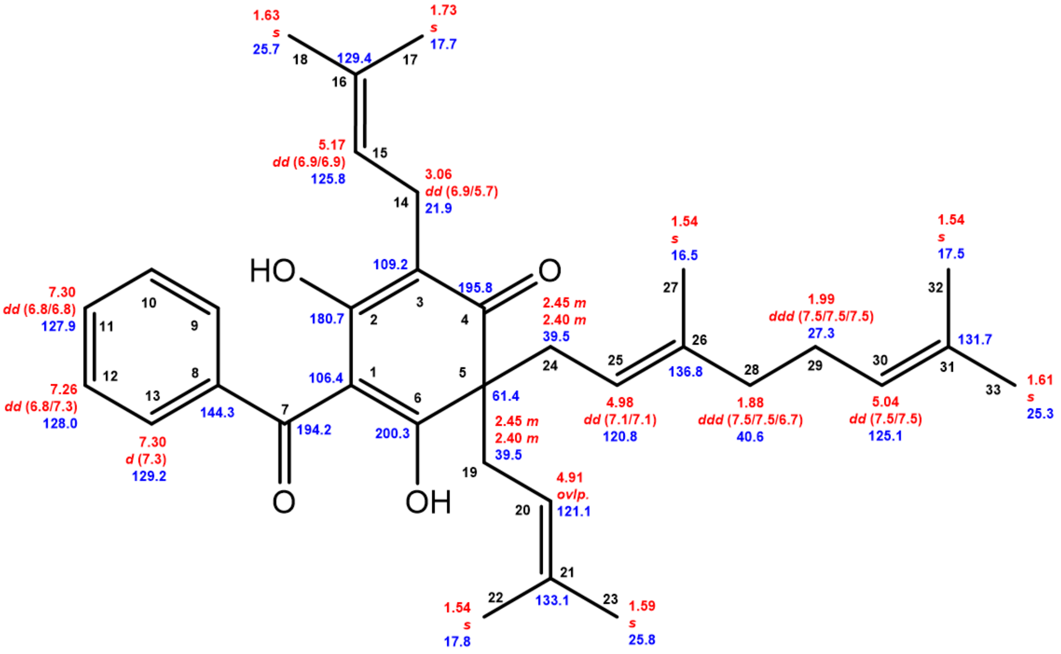

Supplementary Fig. 12 | Chemical shifts of isolated kolanone **2**. Red: ^{1}H chemical shifts (δ ppm, *mult*., $^{3}J_{HH}$ in Hz). Blue: ^{13}C chemical shifts (δ ppm).

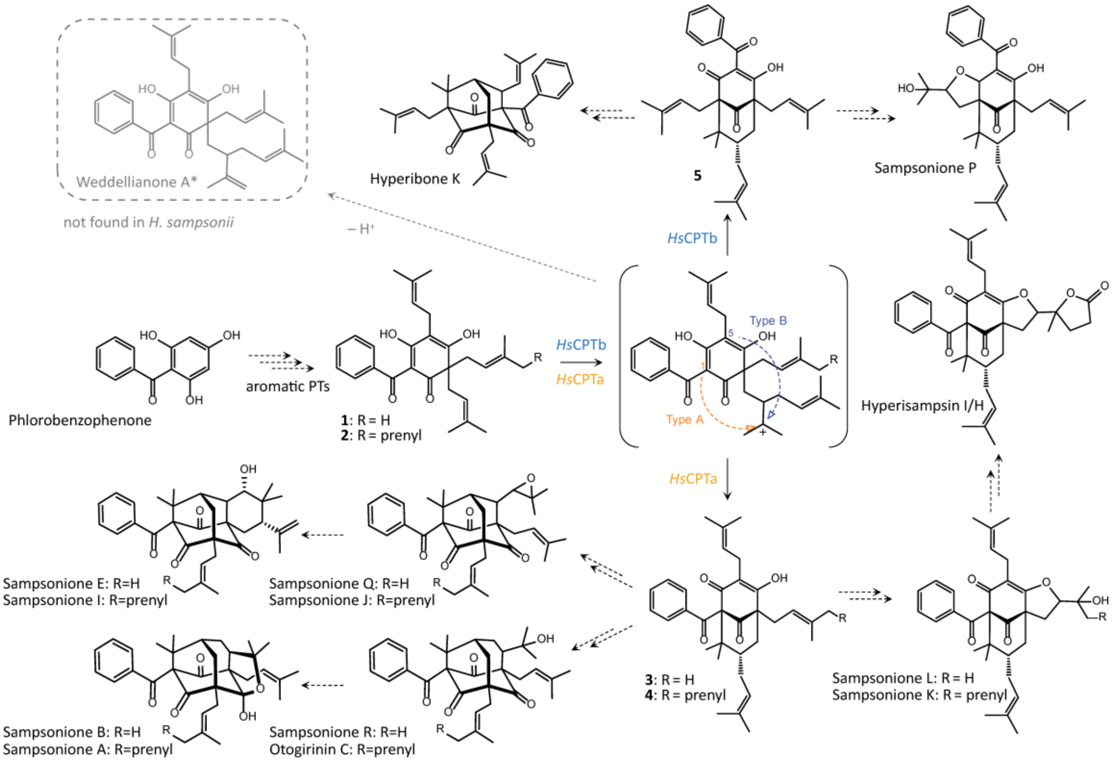

Supplementary Fig. 13 | Illustration of the *Hs*CPTa and *Hs*CPTb catalysed reactions in the context of PPAP biosynthesis. Operating at the branching point between mono- and bicyclic phlorbenzophenone derivatives, *Hs*CPTa and *Hs*CPTb play a central role in the assembly of the bridged bicyclononane core. The identified enzymatic products **3**, **4** and **5** are proposed precursors to many of the specialized metabolites in *H. sampsonii*.

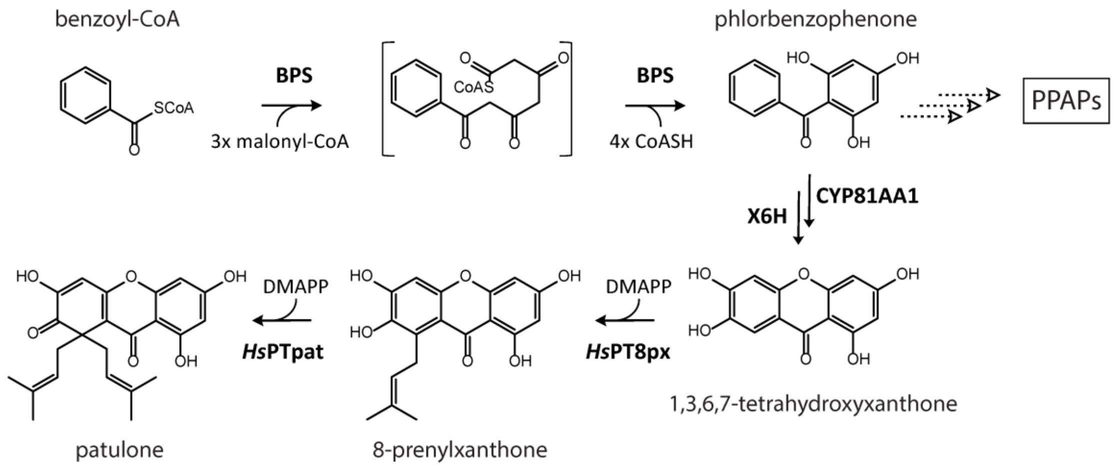

Supplementary Fig. 14 | Biosynthesis of prenylated xanthones in *H. sampsonii*. The xanthone scaffold is closely related to the acylphloroglucinol structure of phlorbenzophenone. Two aromatic PTs, *Hs*PT8px and *Hs*PTpat, are responsible for the sequential diprenylation of 1,3,6,7-tetrahydroxyxanthone in *H. sampsonii*[1] and used as BLAST probes in this study. BPS, benzophenone synthase; CYP81AA1, trihydroxyxanthone synthase; X6H, xanthone 6-hydroxylase.

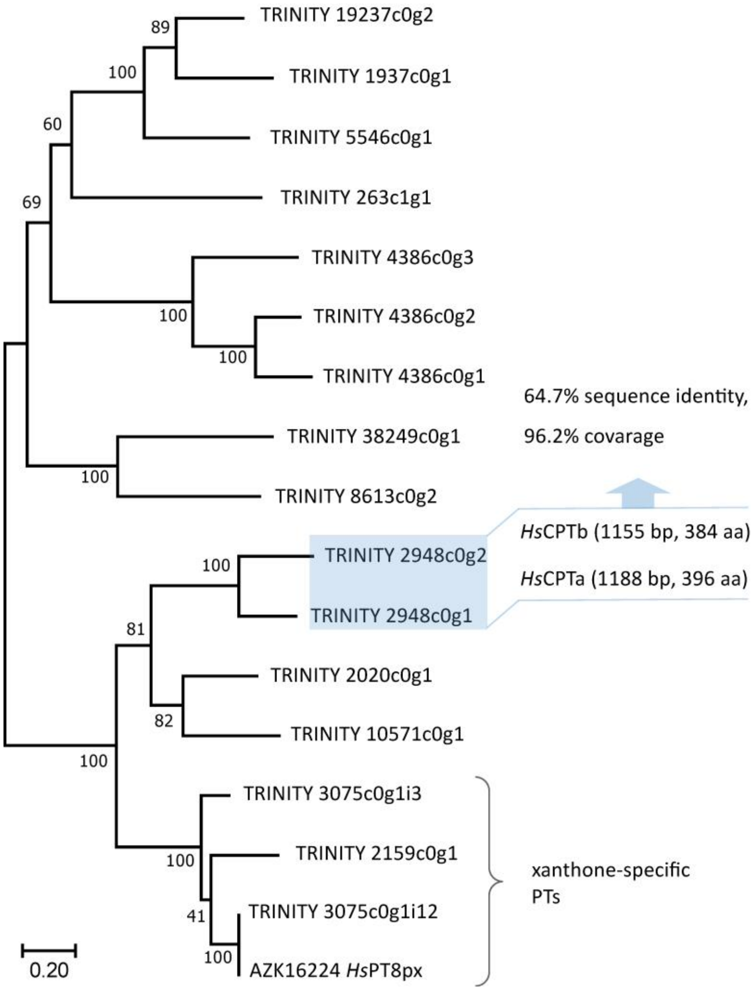

Supplementary Fig. 15 | Phylogenetic estimation of putative PTs according to the Maximum Likelihood method. Homologs were identified by translated nucleotide BLAST search (tblastn), based on similarity to the characterized enzyme *Hs*PT8px.

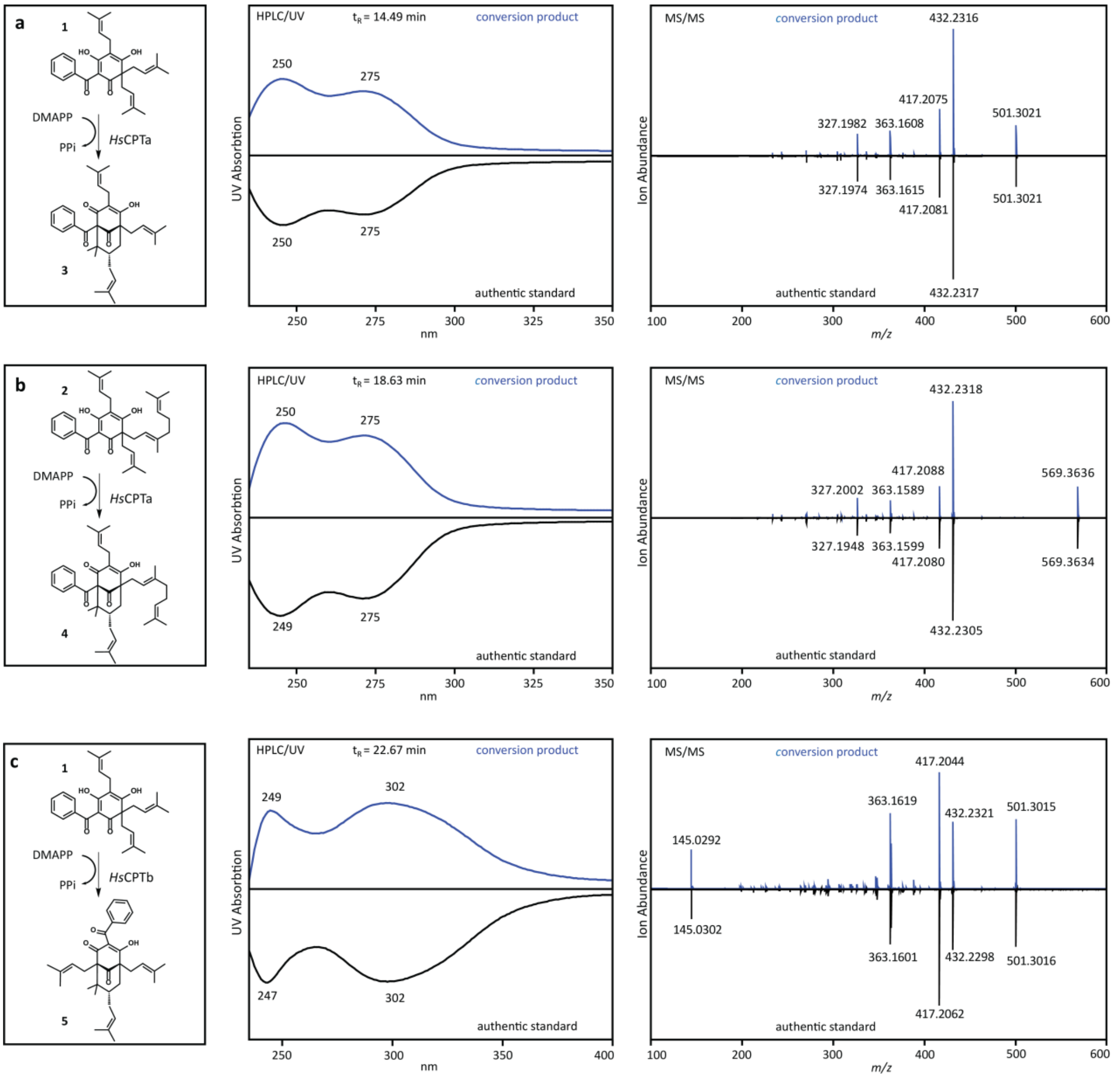

Supplementary Fig. 16 | Confirmation of product identities. Enzymatically formed products and structure-elucidated plant components are compared by their retention time, UV spectrum and tandem MS fragmentation pattern. **a**, 7-*epi*-nemorosone **3**; **b**, nemosampsone **4**; **c**, 7-*epi*-clusianone **5**.

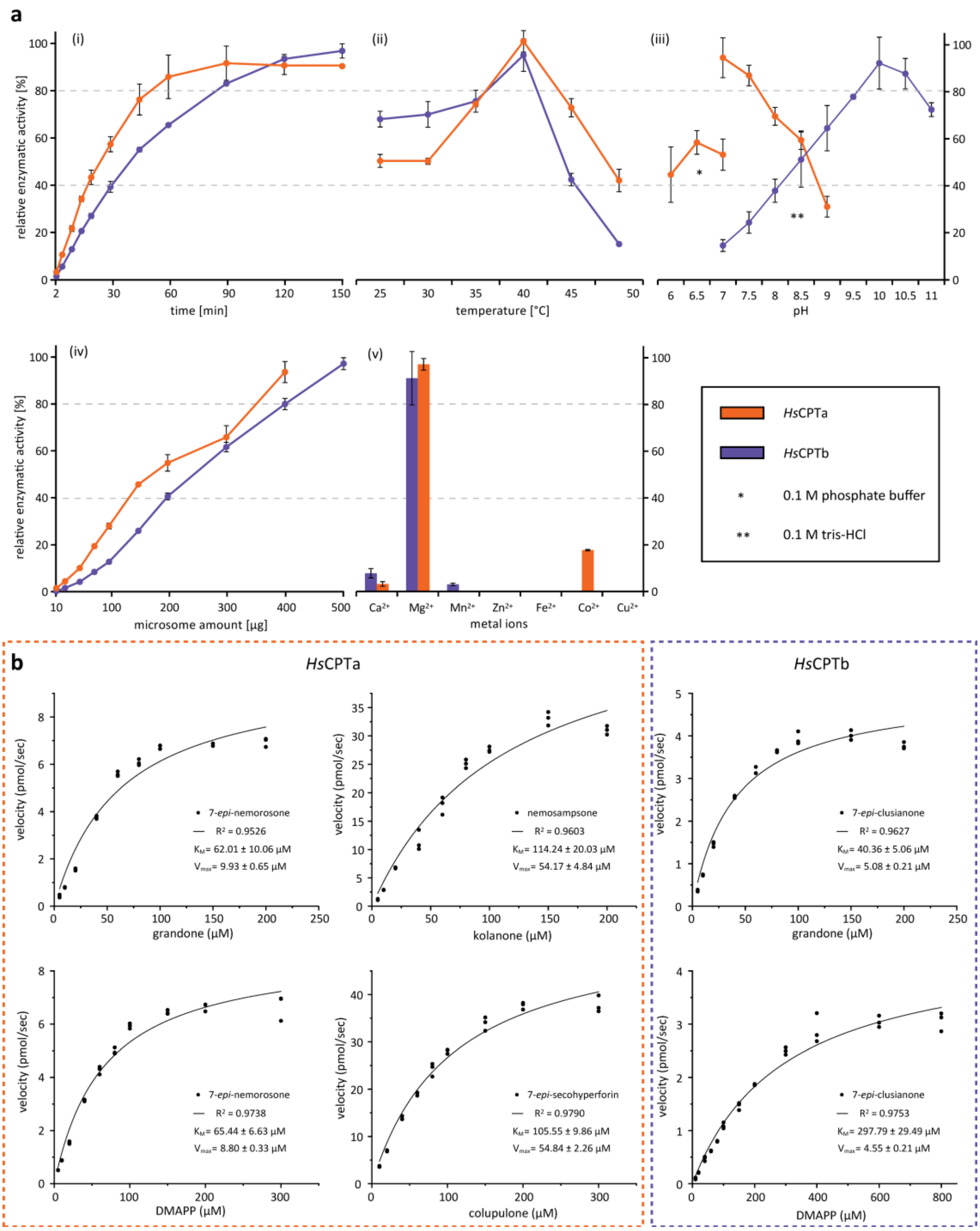

Supplementary Fig. 17 | Characterization of *Hs*CPTa and *Hs*CPTb. a, Optimization of assay parameters. (i) Incubation periods of 10 and 30 minutes for *Hs*CPTa and *Hs*CPTb, respectively, were in the linear range. (ii)–(iii) The temperature optimum was at 40°C for both enzymes and the pH optimum was at 7 and 10 for *Hs*CPTa and *Hs*CPTb, respectively. (iv) Reactions were linearly dependent on the enzyme concentration. (v) The enzymes were almost exclusively active in coordination with magnesium as the metal ion. **b,** Michaelis-Menten kinetics of *Hs*CPTa and *Hs*CPTb, determined at DMAPP saturation for the acceptor substrates grandone, kolanone and colupulone. Kinetic parameters for DMAPP in *Hs*CPTa- and *Hs*CPTb-catalysed reactions were determined at grandone saturation. Data are means ± SD (n=3). K_M, Michaelis-Menten constant; V_{max}, maximum velocity.

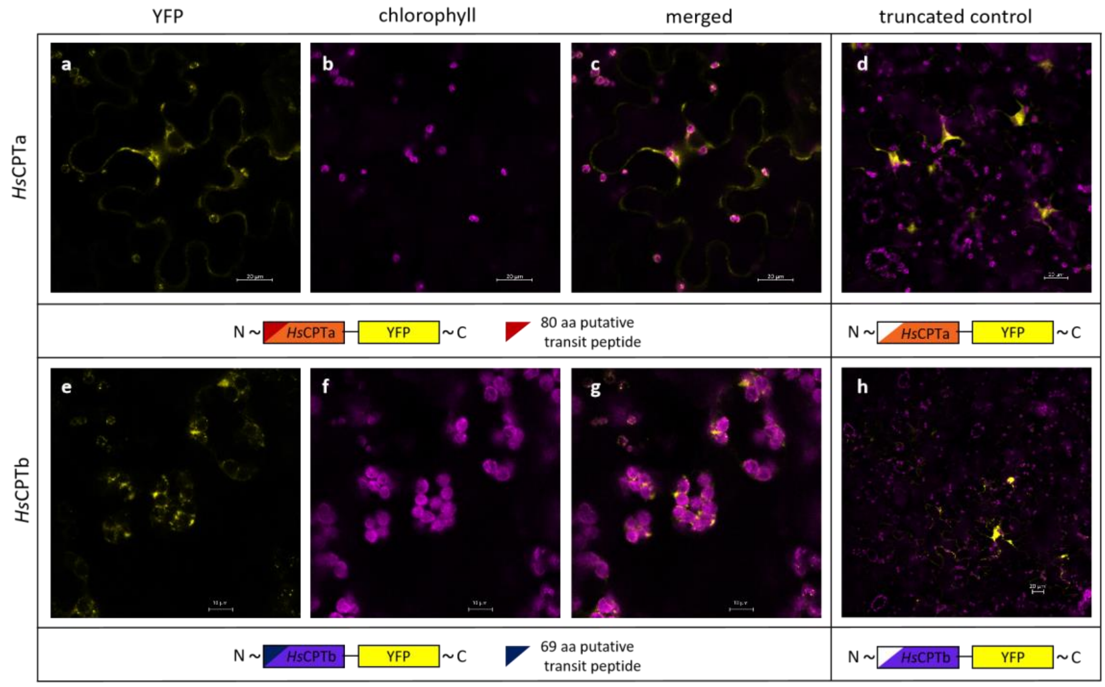

Supplementary Fig. 18 | Localization of *Hs*CPTa and *Hs*CPTb in transiently transformed *Nicotiana benthamiana* leaves. Translational fusion constructs with yellow fluorescent protein (YFP) attached to the C-terminus of *Hs*CPTa **(a–c)** or *Hs*CPTb **(e–g)** emitted signals matching the periphery of the chlorophyll autofluorescence, suggesting their presence inside the chloroplast envelope. Truncation of N-terminal leader sequences confirmed their involvement in the functional localization. The absence of the leader sequence led to accumulation in the vicinity of nucleus/endoplasmic reticulum **(d)** or the cytosol and clustered vesicles **(h)**.

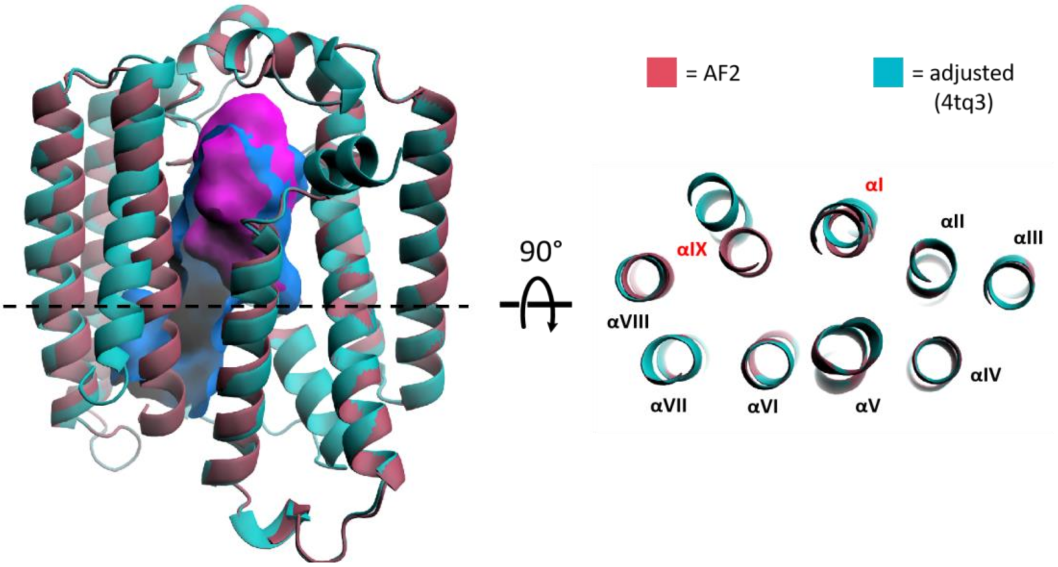

Supplementary Fig. 19 | Structure of the initial AF2-generated *Hs*CPTa model (red) superimposed by the adjusted model with a larger binding cavity in accordance to 4tq3 (turquoise). The two relevant modified helices αI and αIX are highlighted (right panel).

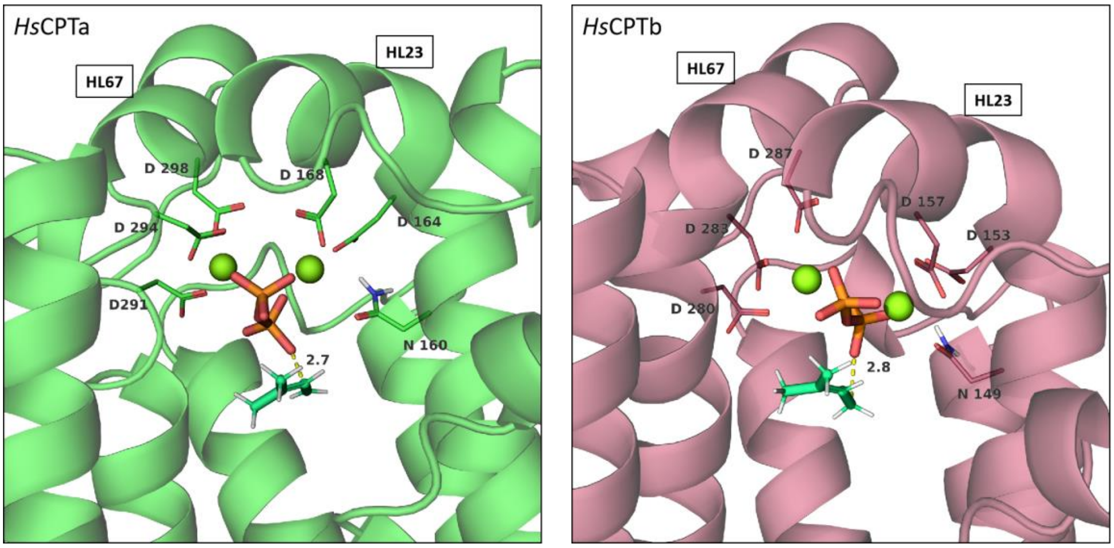

Supplementary Fig. 20 | Simulated binding of the Mg^{2+} ions (spheres) and DMAPP to the aspartate-rich regions of *Hs*CPTa and *Hs*CPTb located in the loops between the helices αI–II and αVI–αVII, using 4qt3 as the reference structure. The isoprenyl cation is dissociated from the pyrophosphate within a distance of 2.8 Å (dashed lines).

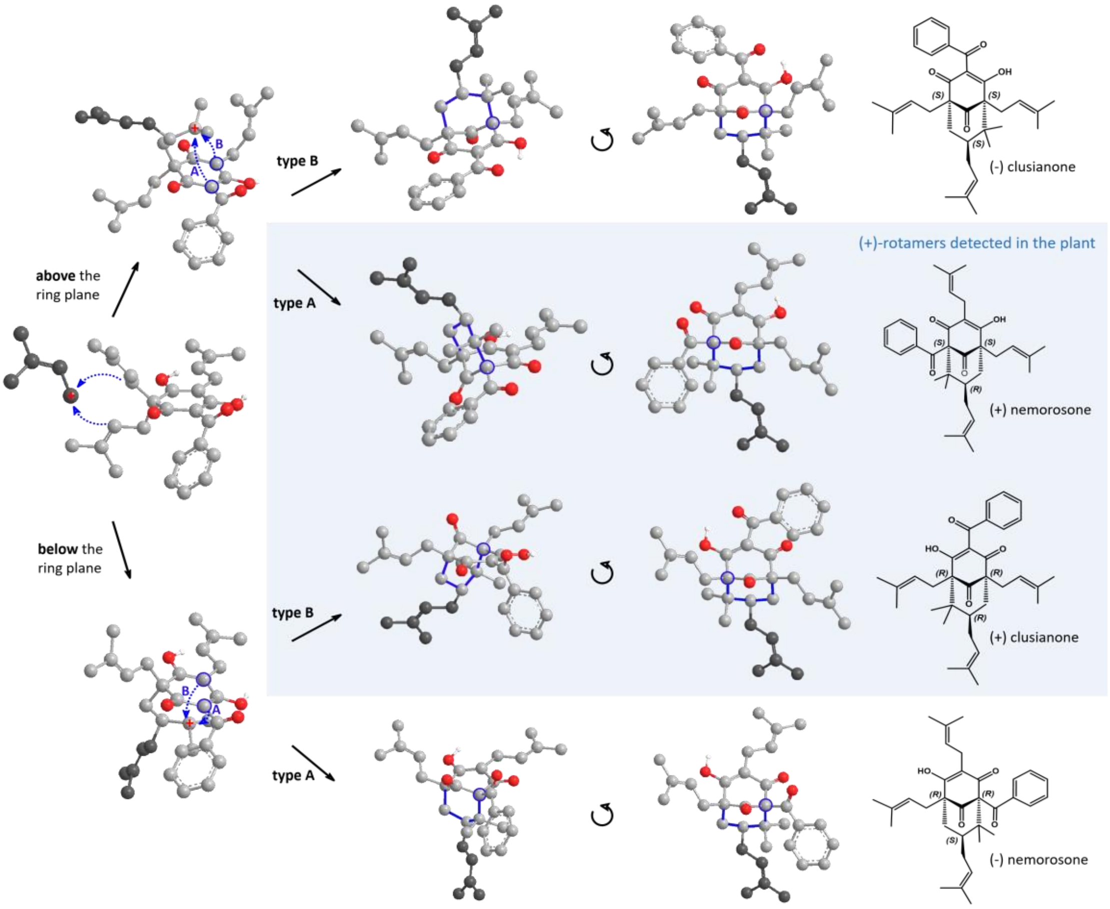

Supplementary Fig. 21 | Formation of type A and B enantiomers with the same optical rotation arises from activation by prenyl transfer of opposing C-3 *gem*-prenyl residues of the acceptor substrate, which undergo cyclization above and below the phloroglucinol ring plane.

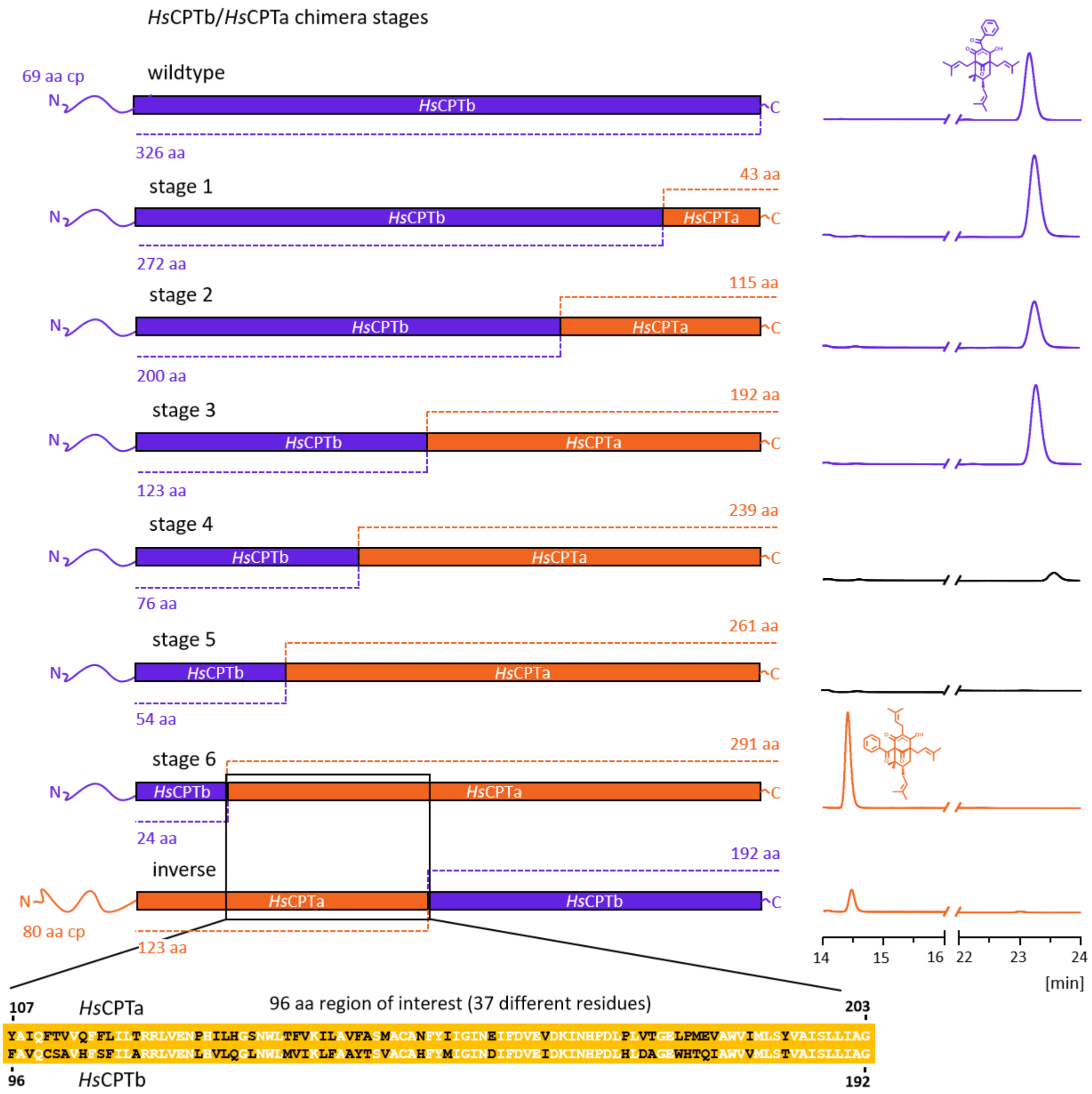

Supplementary Fig. 22 | Analysis of *Hs*CPTb/*Hs*CPTa C-terminal exchange chimeras. Stages 1–3 did not affect the activity. A loss of function was detected after the exchange of 239 and 261 amino acids (stages 4 and 5, respectively). Activity was restored but with switched specificity after substitution of 291 amino acids (stage 6). The inverse mutant of stage 3 confirmed that 123 N-terminal residues of *Hs*CPTa and *Hs*CPTb are sufficient to yield the specific regiomers. Further analysis therefore focused on the highlighted 96 amino acids region, which comprised a total of 37 different residues (black). cp, chloroplast transit peptide.

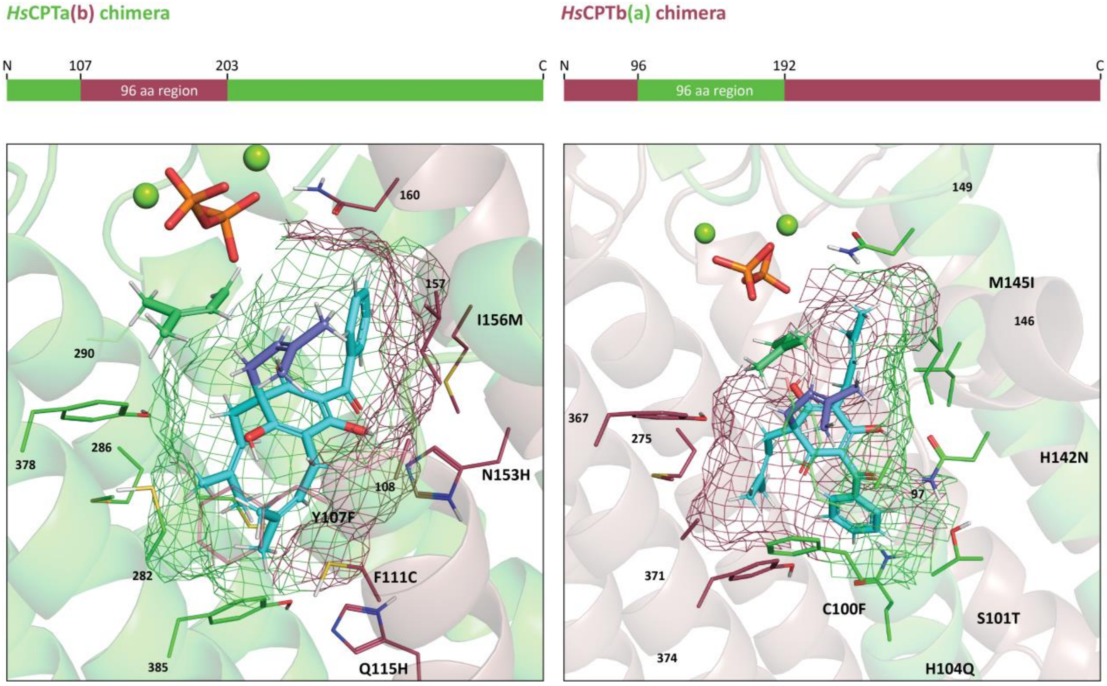

Supplementary Fig. 23 | Molecular modelling of *Hs*CPTa/b chimeras and substrate docking. Reciprocal mutants with exchanged 96 amino acids region showed inverted substrate binding modes, rationalising their switched regiospecificities. The docked substrate conformations were similar to those obtained in simulations with the wildtype enzymes. Residues at the interface of the binding pocket outside the exchanged regiospecificity-determining region appear to be highly conserved. Residues derived from *Hs*CPTa and *Hs*CPTb are shown in green and red, respectively, in each representation.

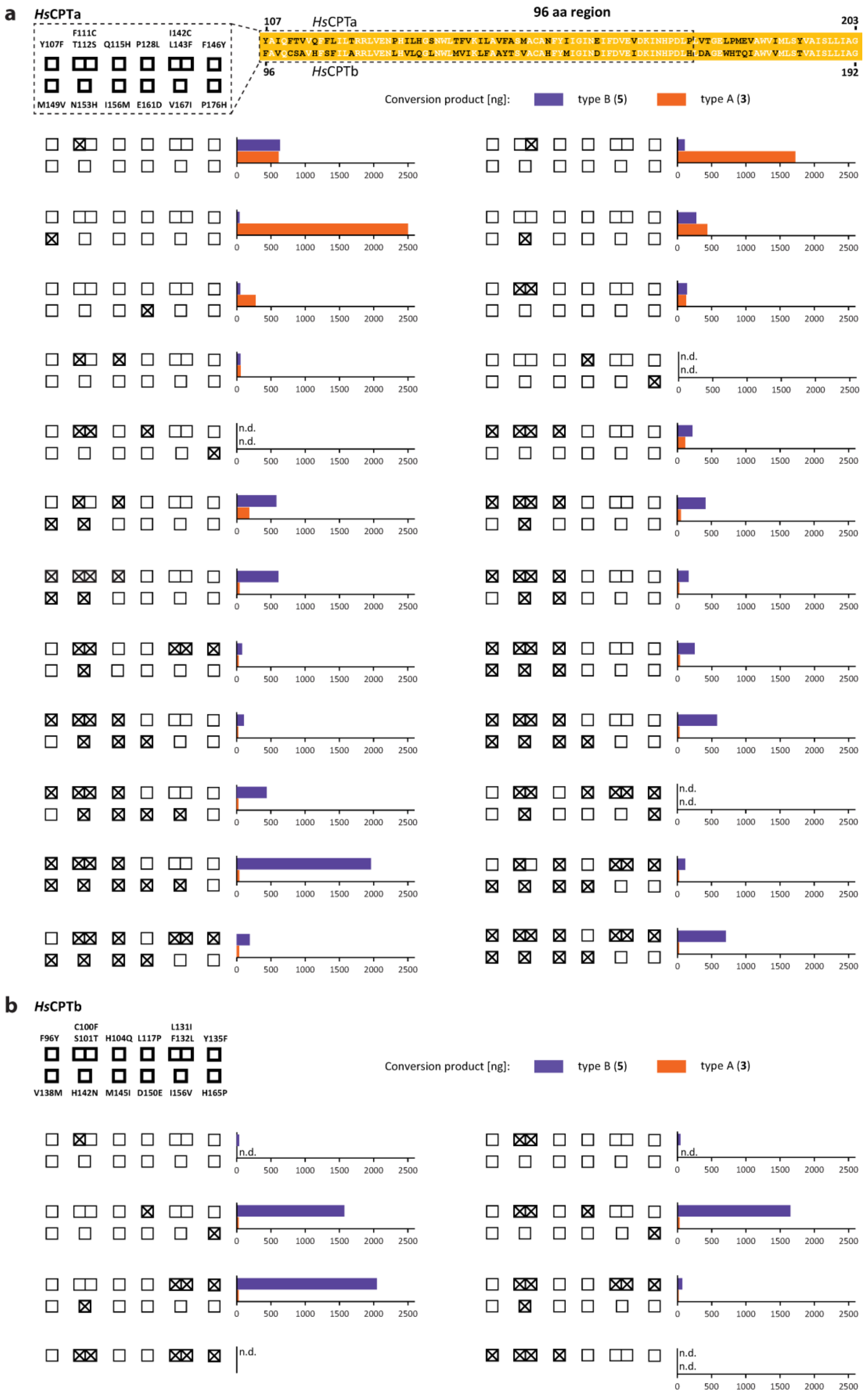

Supplementary Fig. 24 | Summary of tested mutants. Different point mutations and combinations of reciprocal amino acid substitutions were generated for *Hs*CPTa **(a)** and *Hs*CPTb **(b)**, followed by activity screening. Yields of type A and B products are indicated, as obtained from single standardized screening reactions (n=1). n.d., not detected.

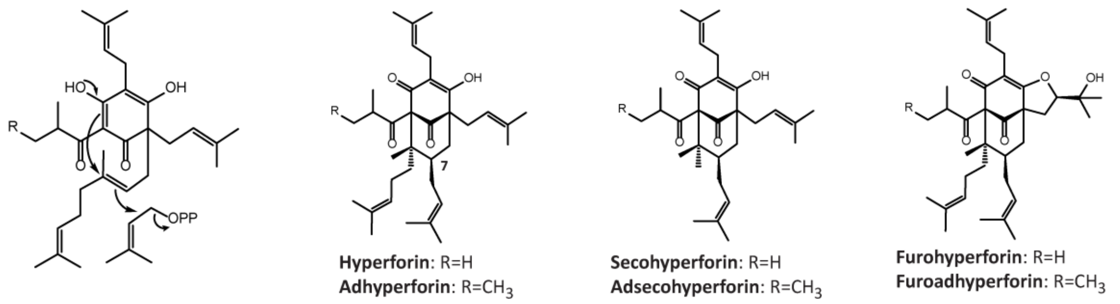

Supplementary Fig. 25 | Examples of PPAP structures with aliphatic acyl groups typically found in *H. perforatum* (St. John's wort) extract. The prenylative cyclization reaction that likely forms the hyperforin scaffold is shown on the left. C-7 *exo* stereochemistry was observed for all elucidated compounds.

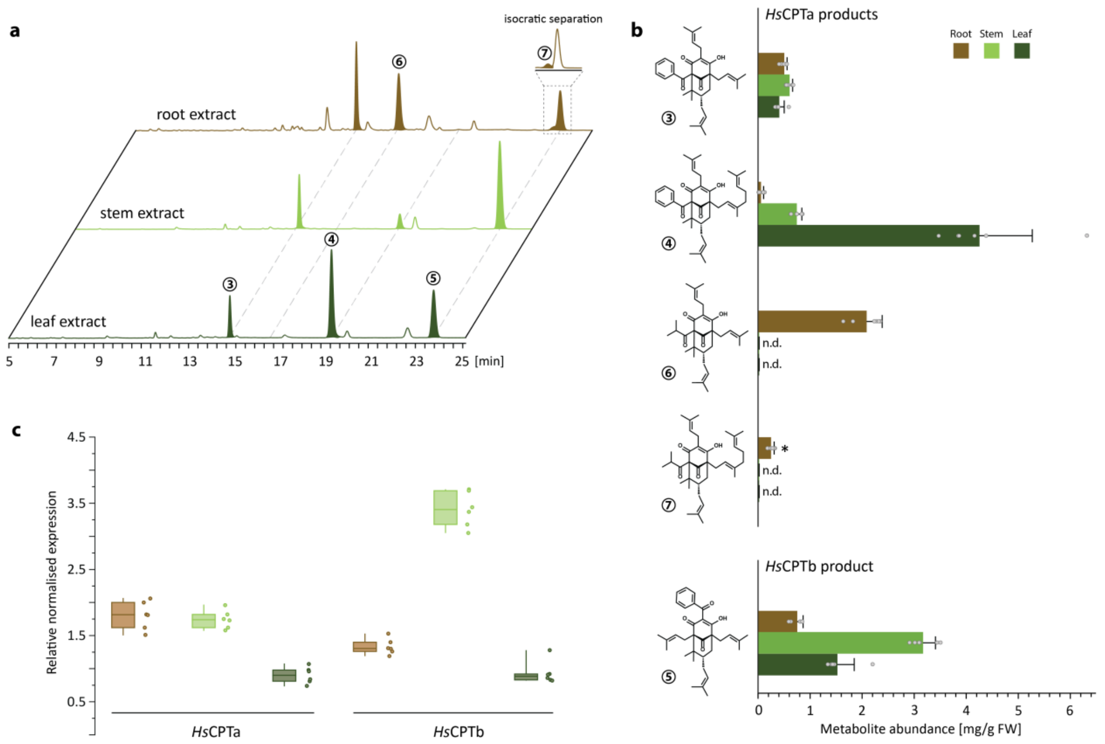

Supplementary Fig. 26 | Metabolite and gene expression levels. a, b Metabolite levels detected in dichloromethane extracts of root, stem, and leaf samples. **c** Relative gene expression of *Hs*CPTa and *Hs*CPTb in root, stem, and leaf tissues. Quantification was based on two biological replicates (n=6). n.d., not detected. Metabolite abundance data are geometric means ± SD. Box-plot bars represent median and upper/lower quartiles. Whiskers represent 1.5× interquartile range. *, The compound **7** content was estimated using the calibration curve of compound **6**.

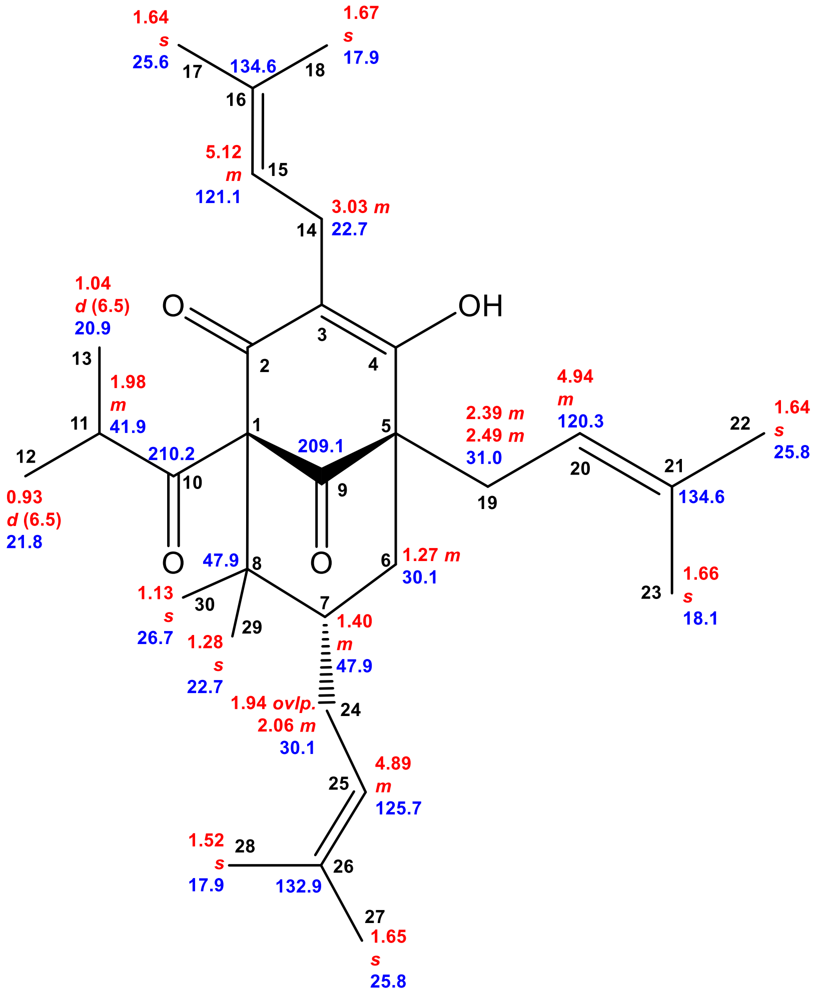

Supplementary Fig. 27 | Chemical shifts of isolated 7-*epi*-secohyperforin **6**. Red: ^{1}H chemical shifts (δ ppm, *mult*., $^{3}J_{HH}$ in Hz). Blue: ^{13}C chemical shifts (δ ppm).

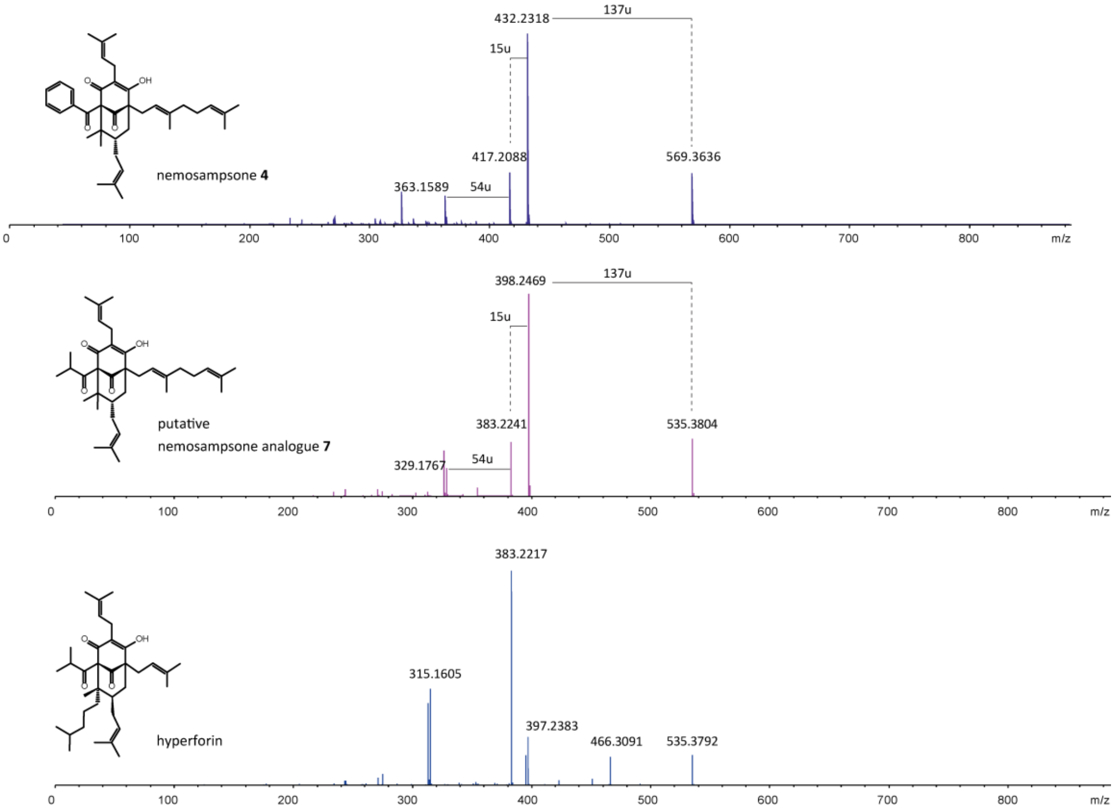

Supplementary Fig. 28 | Alignment of MS/MS spectra. The fragmentation pattern of the putative nemosampsone analogue **7** is shown in comparison to those of nemosampsone **4** and hyperforin. Spectral data were obtained at 35 eV collision energy.

Supplementary Tables

Supplementary Table 1 | ^{1}H and ^{13}C shifts, J_{HH} coupling constants and observed 2D-correlations of compound **4**. Asterisks indicate the lacking NMR signals for highly keto/enolized positions.

No.	δ_C	δ_H (*J* Hz)	^{1}H–^{1}H COSY	^{1}H–^{13}C HMBC
1	77.3	-		
2	*	-		
3	120.6	-		
4	*	-		
5	59.7	-		
6	40.3	2.22 *d* (13.8)<br>2.11 *dd* (7.2, 13.8)	H-7	C-5, 7, 8, 9, 22, 27
7	49.8	1.51 *dddd* (6.3, 6.3, 7.2, 13.8)	H-6b, 27b	C-28
8	50.4	-		
9	210.1	-		
10	195.0	-		
11	138.2	-		
12	129.6	7.54 *d* (8.0)	H-13	C-10, 14
13	128.7	7.22 *dd* (8.0, 7.4)	H-12, 14	C-11, 15
14	133.0	7.41 *dd* (7.4, 7.4)	H-13, 15	C-12, 16
15	128.7	7.22 *dd* (8.0, 7.4)	H-14, 16	C-11, 13
16	129.6	7.54 *d* (8.0)	H-15	C-12, 14
17	22.7	3.06 *m*	H-18	C-3, 19
18	121.4	5.05 *dd* (7.3, 7.3)	H-17	C-20, 21
19	133.7	-		
20	18.0	1.60 *s*		C-19, 21
21	26.0	1.60 *s*		C-19, 20
22	31.1	2.53 *br d* (6.7)	H-23	C-5, 6, 9, 23, 24
23	120.2	5.00 *dd* (6.7, 6.7)	H-22	C-5, 22, 25, 26
24	138.9	-		
25	17.0	1.68 *s*		C-23, 24, 26
26	41.0	1.92 *dd* (7.7, 7.7)	H-1'	C-23, 24, 25, 1', 2'
27	31.0	2.11 *dd* (6.3, 6.3)<br>2.09 *dd* (6.3, 13.8)	H-28	C-7, 28, 29
28	126.3	4.93 *dd* (6.3, 6.3)	H-27	C-30, 31
29	133.1	-		
30	18.2	1.56 *s*		C-28, 29, 31
31	26.0	1.67 *s*		C-28, 29, 30
32	23.3	1.44 *s*		C-1, 7, 8, 33
33	27.3	1.31 *s*		C-1, 7, 8, 32
1'	27.9	2.04 *dd* (7.1, 7.7)	H-26, 2'	C-24, 26, 2'
2'	125.2	5.10 *dd* (7.1, 7.1)	H-1'	C-4', 5'
3'	132.3	-		
4'	17.9	1.59 *s*		C-2', 3', 5'
5'	25.9	1.65 *s*		C-2', 3', 4'

Supplementary Table 2 | Empirical determination of C-7 stereochemistry by comparison of key chemical shifts in accordance to the Grossman-Jacobs rule.

***endo*-type**	$\delta_{H\text{-}6a/b}$	$\delta_{C\text{-}7}$	$\delta_{C\text{-}31/32}$
Compound **3**	2.22, 2.08 (Δδ 0.14)	49.5	23.3, 27.2 (Δδ 3.9)
Compound **4**	2.22, 2.11 (Δδ 0.11)	49.8	23.3, 27.3 (Δδ 4.0)
Compound **5**	2.16, 2.11 (Δδ 0.05)	47.2	22.7, 26.8 (Δδ 4.1)
O-methyl-7-*epi*-nemorosone[2]	2.18, 2.12 (Δδ 0.06)	48.5	23.8, 27.2 (Δδ 3.4)
7-*epi*-clusianone[3]	2.26, 2.22 (Δδ 0.04)	46.3	22.5, 27.0 (Δδ 4.5)
hypersampsone T[4]	2.15, 2.05 (Δδ 0.10)	48.9	23.6, 27.4 (Δδ 3.8)
hypersampsone H[5]	2.18, 2.06 (Δδ 0.12)	48.7	23.3, 27.2 (Δδ 3.9)
***exo*-type**	$\delta_{H\text{-}6a/b}$	$\delta_{C\text{-}7}$	$\delta_{C\text{-}31/32}$
nemorosone[6]	1.97, 1.42 (Δδ 0.55)	42.1	15.6, 23.2 (Δδ 7.6)
clusianone[3]	2.17, 1.62 (Δδ 0.55)	42.3	16.2, 22.6 (Δδ 6.4)
O-methyl-hydroxynemorosone[7]	1.94, 1.42 (Δδ 0.52)	42.4	16.1, 24.4 (Δδ 8.3)
O-methylchamone I[8]	1.92, 1.45 (Δδ 0.47)	42.3	16.4, 24.7 (Δδ 8.3)

Supplementary Table 3 | Comparison of ^{1}H and ^{13}C shifts and J_{HH} coupling constants reported for secohyperforin[9] and extracted from compound **6**. The chemical shifts important for determination of C-7 stereochemistry according to the Grossman-Jacobs rule are highlighted in red.

**No.**	**Reference** δ_C	**Reference** δ_H (*J* Hz)	**Compound 6** δ_C	**Compound 6** δ_H (*J* Hz)
1	n.d.	-	n.d.	-
2	n.d.	-	n.d.	-
3	n.d.	-	n.d.	-
4	n.d.	-	n.d.	-
5	n.d.	-	n.d.	-
6	n.d.	1.39 *m*<br>1.85 *m*	30.1	**1.27 *m***
7	43.8	1.49 *m*	**47.9**	1.40 *m*
8	46.8	-	47.9	-
9	n.d.	-	209.1	-
10	210.6	-	210.2	-
11	41.5	2.15 *br*	41.9	1.98 *m*
12	21.6	0.94 *d* (6.7)	21.8	0.93 *d* (6.5)
13	20.9	1.03 *d* (6.5)	20.9	1.04 *d* (6.5)
14	22.2	3.06 *d* (6.8)	22.7	3.03 *m*
15	121.8	5.04 *m*	121.1	5.12 *m*
16	133.9	-	134.6	-
17	25.6	1.63 *s*	25.6	1.64 *s*
18	17.9	1.67 *s*	17.9	1.67 *s*
19	30.1	2.40 *dd* (7.1, 14.9)<br>2.47 *br*	31.0	2.39 *m*<br>2.49 *m*
20	120.6	4.93 *m*	120.3	4.94 *m*
21	134.4	-	134.6	-
22	25.8	1.61 *s*	25.6	1.64 *s*
23	18.0	1.63 *s*	18.1	1.66 *s*
24	27.5	2.07 *ovlp.*	30.1	1.94 *ovlp.*<br>2.06 *m*
25	123.6	4.96 *m*	125.7	4.89 *m*
26	133.8	-	132.9	-
27	25.7	1.64 *s*	25.8	1.65 *s*
28	17.7	1.53 *s*	17.9	1.52 *s*
29	23.6	1.16 *s*	**22.7**	1.28 *s*
30	16.0	0.93 *s*	**26.7**	1.13 *s*

Supplementary Table 4 | FPKM values in various *H. perforatum* organs for the transcripts of hpa_locus_470_iso_1_len_1384_ver_2 encoding *Hp*CPTa1. Data are from the MPGR RNA-seq database.

**Organ**	**FPKM value**
HPA_AA, pistils, stamens, and sepals of mature flowers	334.47
HPA_AB, whole flower buds	283.53
HPA_AC, fully expanded mature flowers	249.88
HPA_AD, petals of fully expanded mature flowers	25.19
HPA_AE, pistils, stamens, and sepals of flower buds	48.34
HPA_AF, petals of flower buds	84.38
HPA_AI, whole mid aged leaves	15.08
HPA_AJ, whole old leaves	7.46
HPA_AK, whole young leaves	47.33
HPA_AL, portion of young leaves with light and dark glands	14.90
HPA_AM, portion of old leaves with only light glands	14.65
HPA_AN, portion of young leaves with only light glands	11.32
HPA_AO, portion of old leaves with light and dark glands	19.42
HPA_AQ, middle aged part of the roots	0.00
HPA_AR, oldest part of the roots	0.66
HPA_AS, dark glands of flower petals	5.09
HPA_AT, flower petals	28.29

Supplementary Table 5 | Primers used for plasmid construction, mutagenesis, and RT-qPCR.

**Primer name**	**5' to 3' sequence**
pESC-Ura cloning	
BamHI_HsCPTa F	GCATGGATCCATGGAGATCTCTCTCTTTCCTTCGC
KpnI_HsCPTa R	ATGCGGTACCCTAGATAAAGGGAAGTAGGATCATGTGGG
BamHI_HsCPTb F	GCATGGATCCATGGAGATTTCCCGATGCTTTCC
KpnI_HsCPTb R	ATGCGGTACCCTAGATGAAAGGAAATAGGATTAAGTGGACTAAATAG
BamHI_HpCPTa1 F	ATTGGATCCATGGAGATCTCTTGCTTTCC
HindIII_HpCPTa1 R	ATTAAGCTTCTAGATGAAGGGAAGTAGGATTAAG
USER cloning	
User_HsCPTa_CY R	GGTTTAA[U]CCGATAAAGGGAAGTAGGATCATG
User_truncHsCPTa F	GGCTTAA[U]ATGGAAGATTCGAAAAAAAGTAATATTGACG
User_HsCPTa F	GGCTTAA[U]ATGGAGATCTCTCTCTTTCCTTC
User_HsCPTb_CY R	GGTTTAA[U]CCGATGAAAGGAAATAGGATTAAGTGG
User_truncHsCPTb F	GGCTTAA[U]ATGGAACTTTCGAAAGAGAGTGATG
User_HsCPTb F	GGCTTAA[U]ATGGAGATTTCCCGATGC
C-terminal exchange	
HsCPTb_a_stage1 R	TKCCCGCRATTAGTAAACTTATYGCYAC
HsCPTb_a_stage2 R	GTCTGGAATATCYTTCAAAAAKCCCATGGTRAAGC
HsCPTb_a_stage3 R	TCCATCTTCCAAGCCTGATAAAGCACG
HsCPTb_a_stage4 R	GAAAGGCATAGACTTTAGTGTGCCCGCGATTAGTAAACTTATTG
HsCPTb_a_stage5 R	CGTCAAAAATCTCATTGATGCCAATCATATAGAAGTGTG
HsCPTb_a_stage6 R	CGTAAATTGTATGGCATATGGCCTTAGAAATCGTAATGC
HsCPTa_b_reverse R	CGAAAGGCACAGACCTAAGTATTCCCGCAATTAGTAAACTTATCG

Supplementary Table 5 continued

Primer name	5' to 3' sequence
site-directed mutagenesis	
HsCPTa_F111C F	TACAATgTACGGTGGTGCAATTCTTCTTAATATTG
HsCPTa_F111C R	ACCGTAcATTGTATGGCATATGGCCTC
HsCPTa_T112S F	TACAATTTtCtGTGGTGCAATTCTTCTTAATATTG
HsCPTa_T112S R	ACaGaAAATTGTATGGCATATGGCCTC
HsCPTa_Q115H F	TGGTGCAtTTCTTCTTAATATTGACAAGAAG
HsCPTa_Q115H R	TATTAAGAAGAAaTGCACCACCGTAcATTG
HsCPTa_P128L F	TCGAGAACttGCACATACTTCACGGATCAAATTG
HsCPTa_P128L R	AGTATGTGCaaGTTCTCGACCAATCTTCTTGTC
HsCPTa_P128L_v2 R	AGTATGTGCaaGTTCTCGACCAATCTTCTTGcC
HsCPTa_M149V F	TTGCAAGTgTaGCTTGTGCAAACTTCTATATAATTGG
HsCPTa_M149V_v2 F	TTGCAAGTgTaGCTTGTGCAcACTTCTATATAATTGG
HsCPTa_M149V_v3 F	acGCAAGTgTaGCTTGTGCAcACTTCTATATAATTGG
HsCPTa_M149V R	GCACAAGCtAcACTTGCAAAAACAGCTAATATTTTG
HsCPTa_M149V_v2 R	GCACAAGCtAcACTTGCgtAAACAGCaAATAgTTTG
HsCPTa_N153H F	CTTGTGCAcACTTCTATATAATTGGCATCAATG
HsCPTa_N153H R	ATAGAAGTgTGCACAAGCCATACTTGC
HsCPTa_I156M F	TTCTATATgATTGGCATCAATGAGATTTTTGACG
HsCPTa_I156M R	CCAATcATATAGAAGTgTGCACAAGCCATAC
HsCPTa_I156M_v2 R	CCAATcATATAGAAGTgTGCACAAGCtAcAC
HsCPTa_E161D F	ATCAATGAtATTTTTGACGTTGAAGTAGACAAG
HsCPTa_E161D R	CGTCAAAAATaTCATTGATGCCAATTATATAGAAG
HsCPTa_E161D_v2 R	CGTCAAAAATaTCATTGATGCCAATcATATAGAAG
HsCPTa_V167I F	ACGTTGAAaTAGACAAGATAAATCATCCCGAC
HsCPTa_V167I R	ATCTTGTCTAtTTCAACGTCAAAAATCTCATTGATG
HsCPTa_V167I_v2 R	ATCTTGTCTAtTTCAACGTCAAAAATaTCATTGATG
HsCPTa_P176H F	GACTTGCaTTTGGTTACGGGAGAATTGC
HsCPTa_P176H R	GTAACCAAAtGCAAGTCGGGATGATTTATCTTG
HsCPTa_CS F	CATACAATgTtCtGTGGTGCAATTCTTCTTAATATTGAC
HsCPTa_CS R	ACCACaGaAcATTGTATGGCATATGGCCTC
HsCPTa_LFY F	AAAcTATTtGCTGTTTacGCAAGTATGGCTTGTGCAAAC
HsCPTa_LFY R	TGCgtAAACAGCaAATAgTTTGACAAATGTGAGCCAATTTG
RT-qPCR	
qPCR_HsCPTa F	ATAGCTGCCATGAGACCTCAGG
qPCR_HsCPTa R	GGTAACGGTCTCTGCTTTGTGC
qPCR_HsCPTb F	TCAGTGCCCACTTCCTGTCATC
qPCR_HsCPTb R	ACTCCTACTCCTGTAAACGCGG
qPCR_actin2 F	CCTGGGTTACCTAGTCGCTTGG
qPCR_actin2 R	CCAAAACTCGGGTGCATCCTTC
qPCR_β-tubulin F	GAATACAAGGCGTGCGAGTCTC
qPCR_β-tubulin R	CTGATCCCACTGCATTGCCTTC

Other NMR spectra

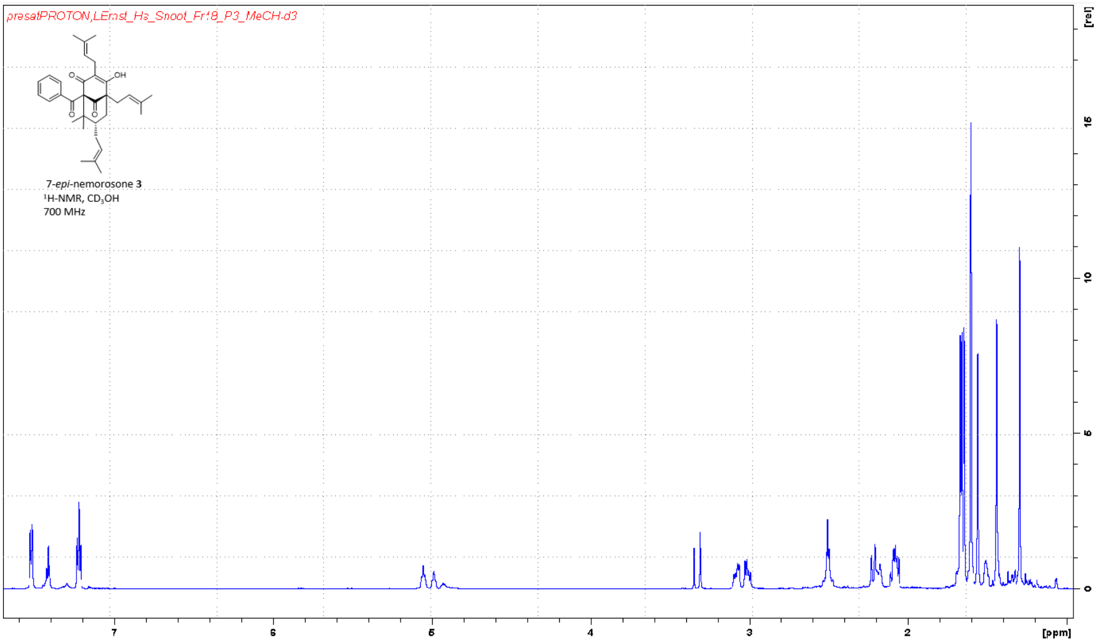

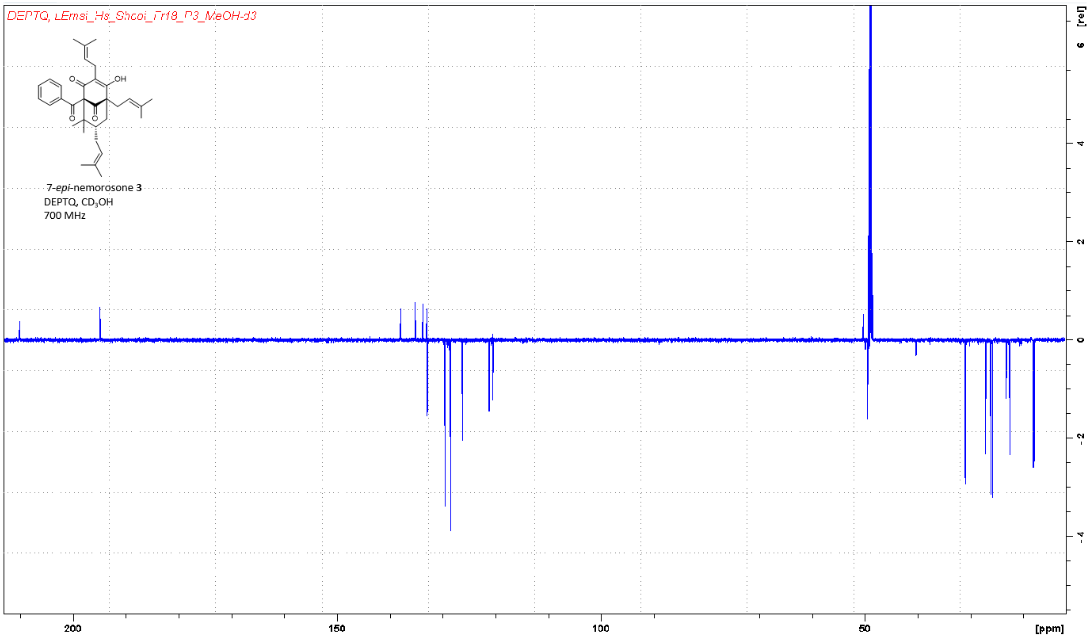

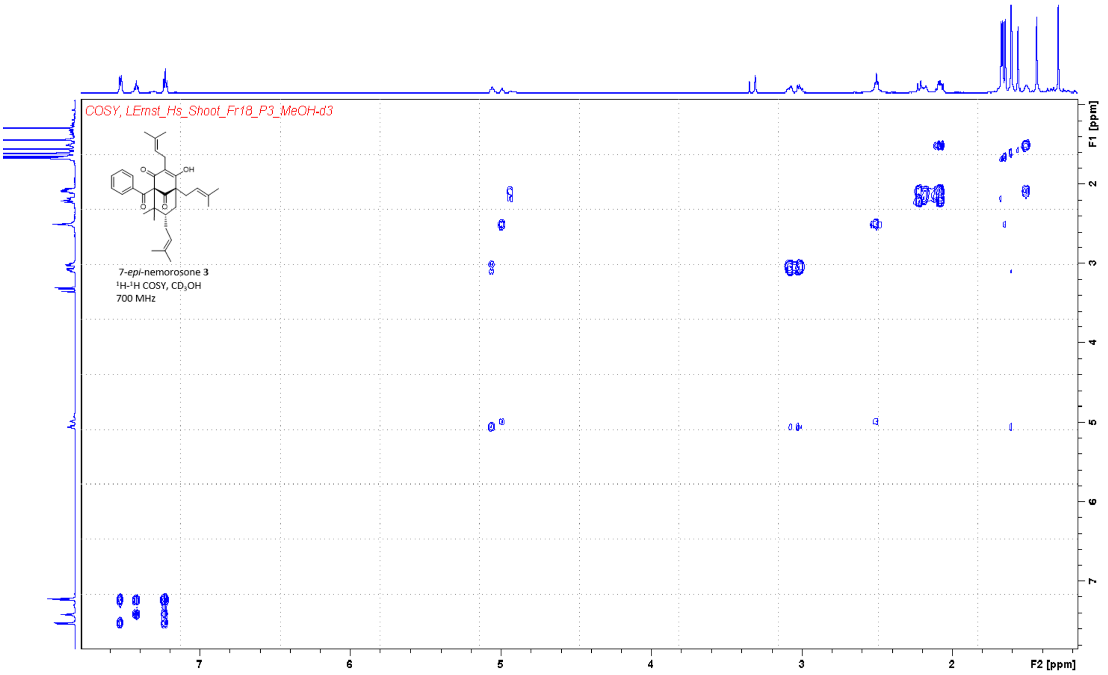
COSY, LErnst_Hs_Shoot_Fr18_P3_MeOH-d3
7-epi-nemorosone 3
¹H-¹H COSY, CD3OH
700 MHz
F1 [ppm]
F2 [ppm]

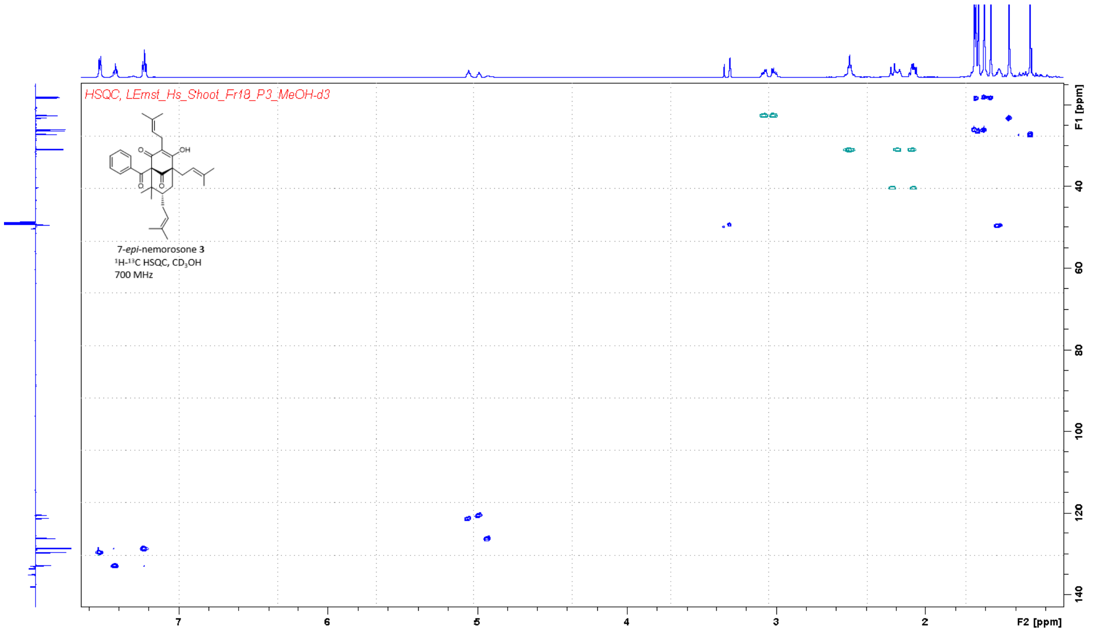
HSQC, LErnst_Hs_Shoot_Fr18_P3_MeOH-d3
7-epi-nemorosone 3
¹H-¹³C HSQC, CD3OH
700 MHz
F1 [ppm]
F2 [ppm]

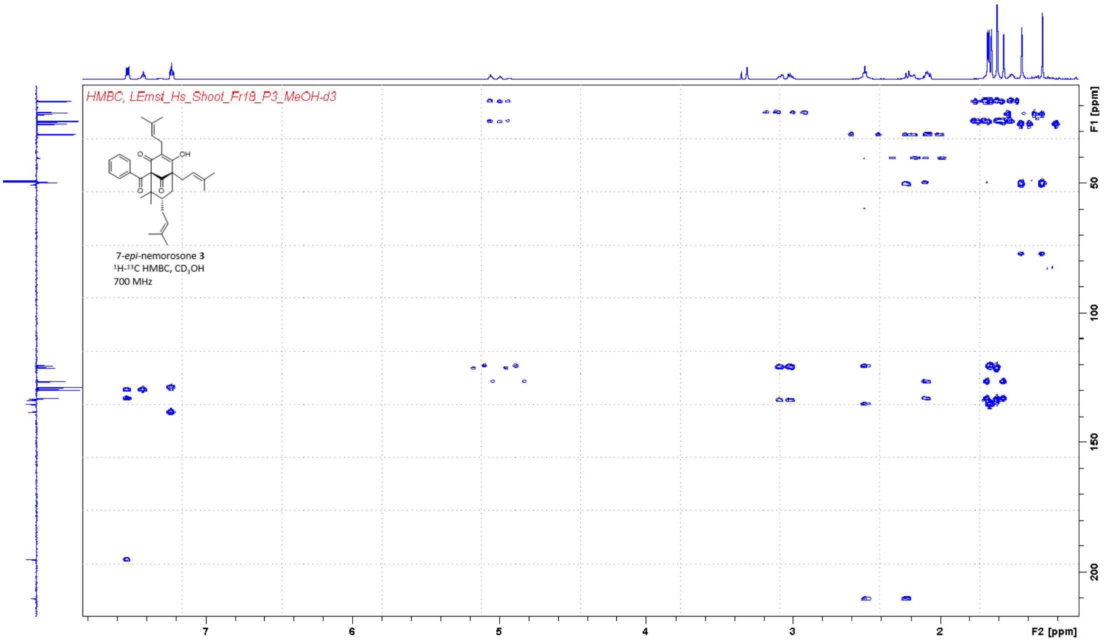
HMBC, LErnst_Hs_Shoot_Fr18_P3_MeOH-d3
7-*epi*-nemorosone 3
¹H-¹³C HMBC, CD₃OH
700 MHz
F1 [ppm]
F2 [ppm]

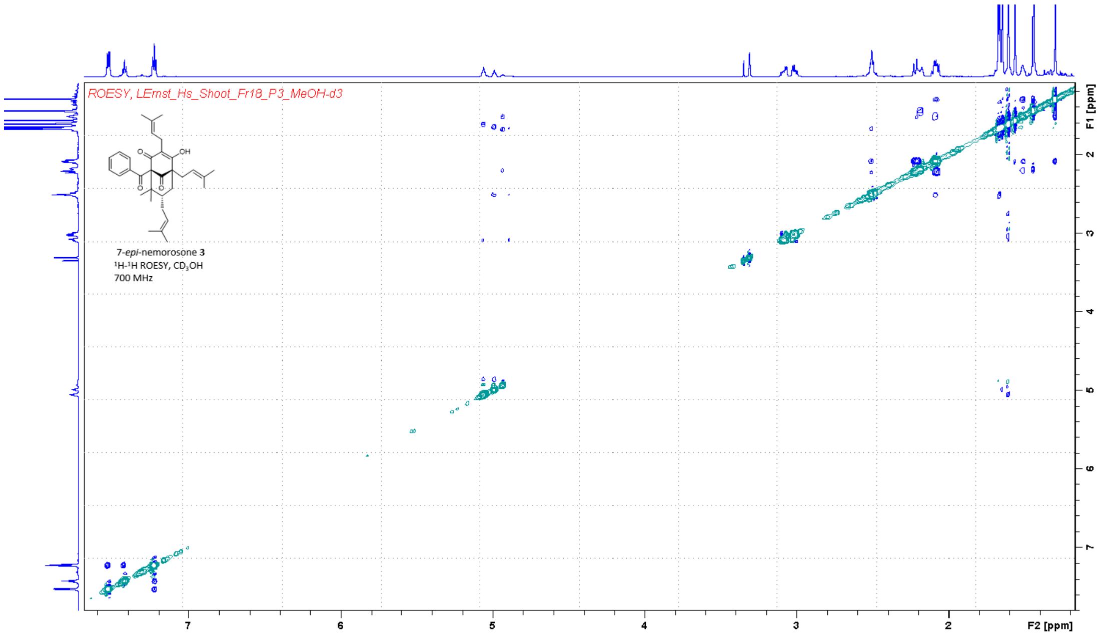
ROESY, LErnst_Hs_Shoot_Fr18_P3_MeOH-d3
7-*epi*-nemorosone 3
¹H-¹H ROESY, CD₃OH
700 MHz
F1 [ppm]
F2 [ppm]

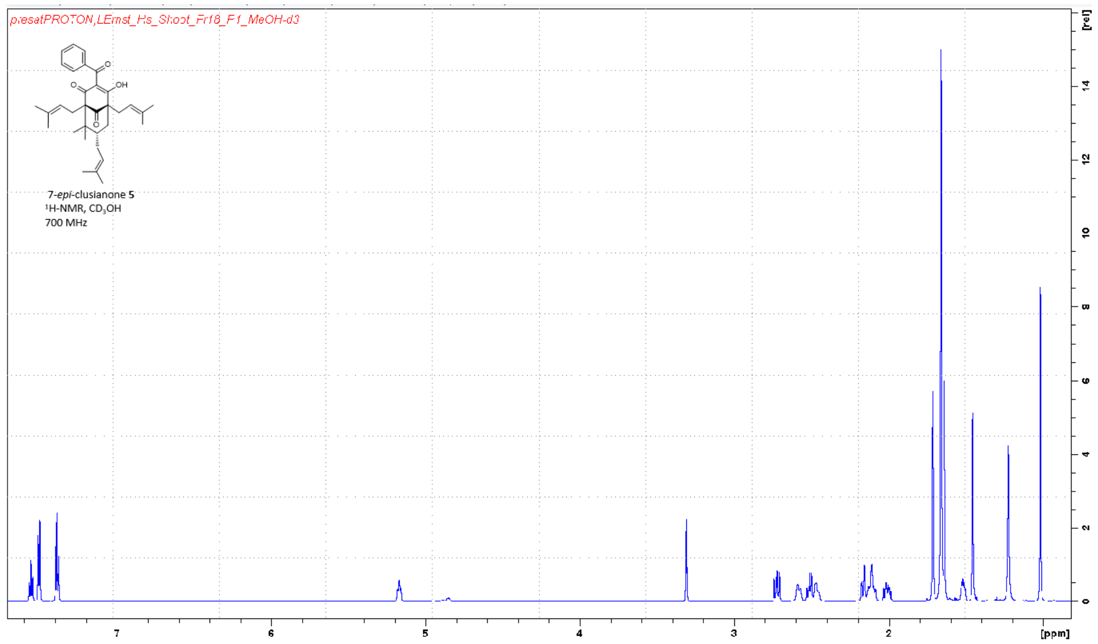
presatPROTON,LErnst_Hs_Shoot_Fr18_F1_MeOH-d3
7-epi-clusianone 5
1H-NMR, CD3OH
700 MHz
[ppm]
[rel]

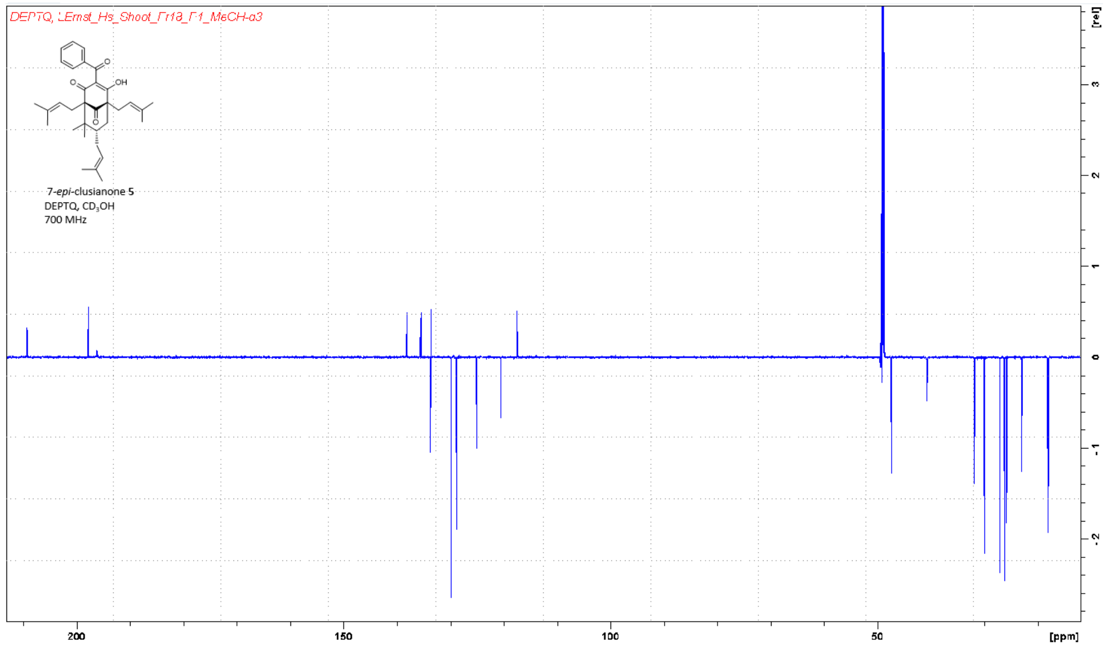
DEPTQ, LErnst_Hs_Shoot_Fr18_F1_MeOH-d3
7-epi-clusianone 5
DEPTQ, CD3OH
700 MHz
[ppm]
[rel]

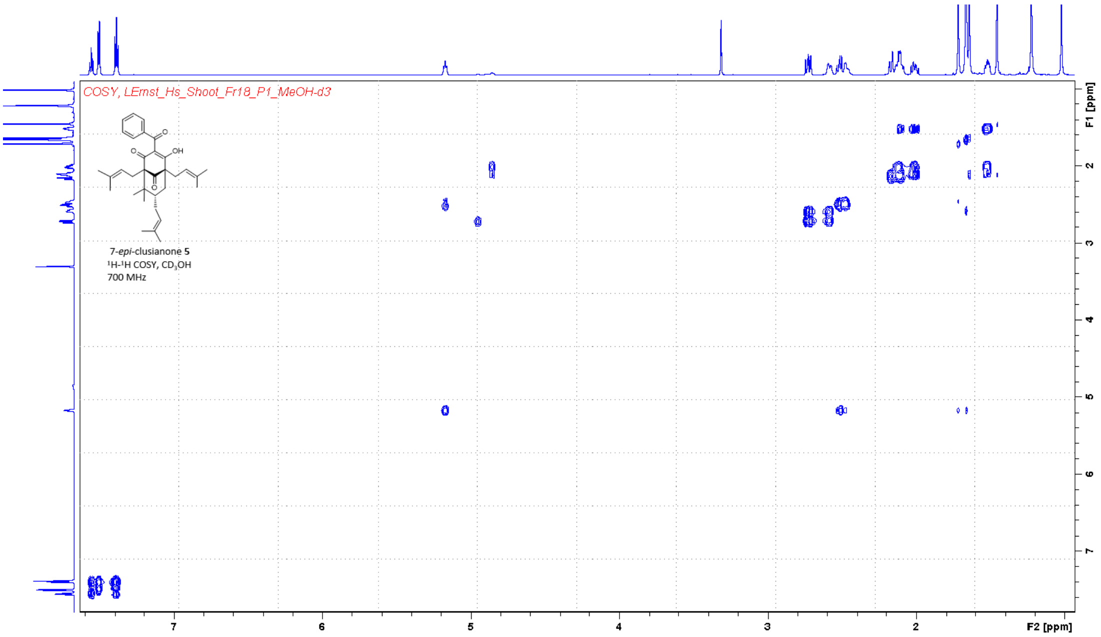
COSY, LErnst_Hs_Shoot_Fr18_P1_MeOH-d3
7-epi-clusianone 5
¹H-¹H COSY, CD3OH
700 MHz
F1 [ppm]
F2 [ppm]

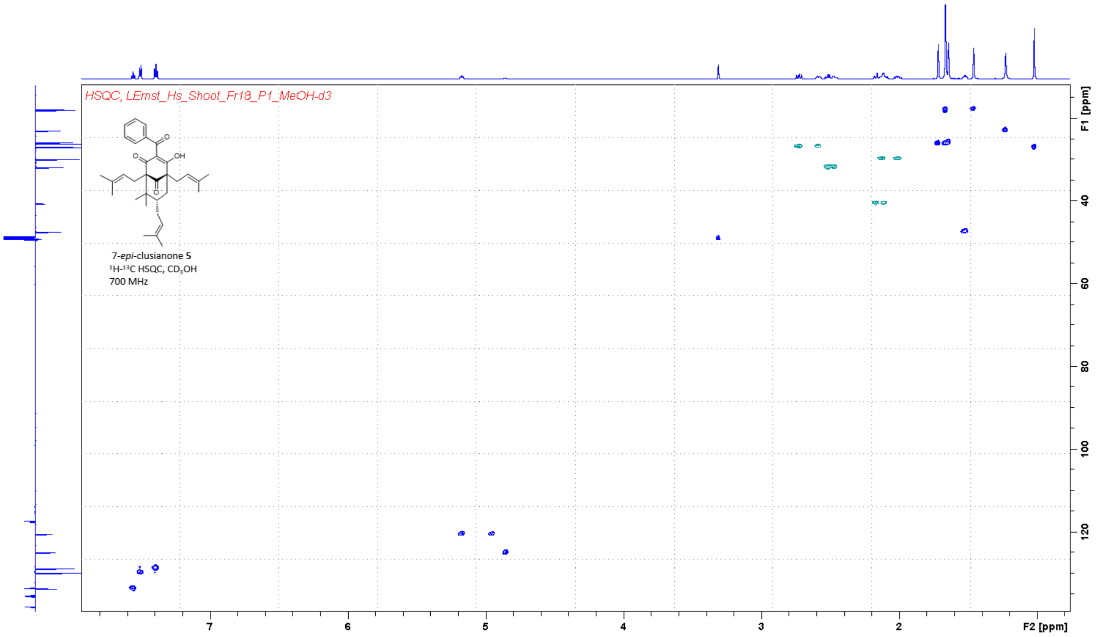
HSQC, LErnst_Hs_Shoot_Fr18_P1_MeOH-d3
7-epi-clusianone 5
¹H-¹³C HSQC, CD3OH
700 MHz
F1 [ppm]
F2 [ppm]

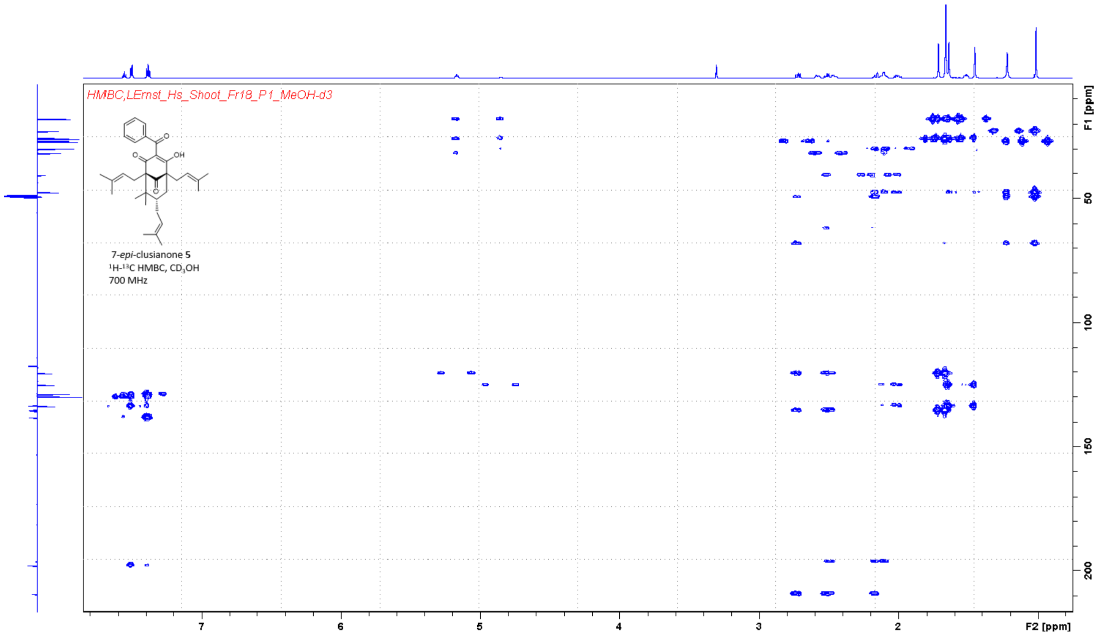
HMBC,LErnst_Hs_Shoot_Fr18_P1_MeOH-d3
7-epi-clusianone 5
1H-13C HMBC, CD3OH
700 MHz
F1 [ppm]
F2 [ppm]

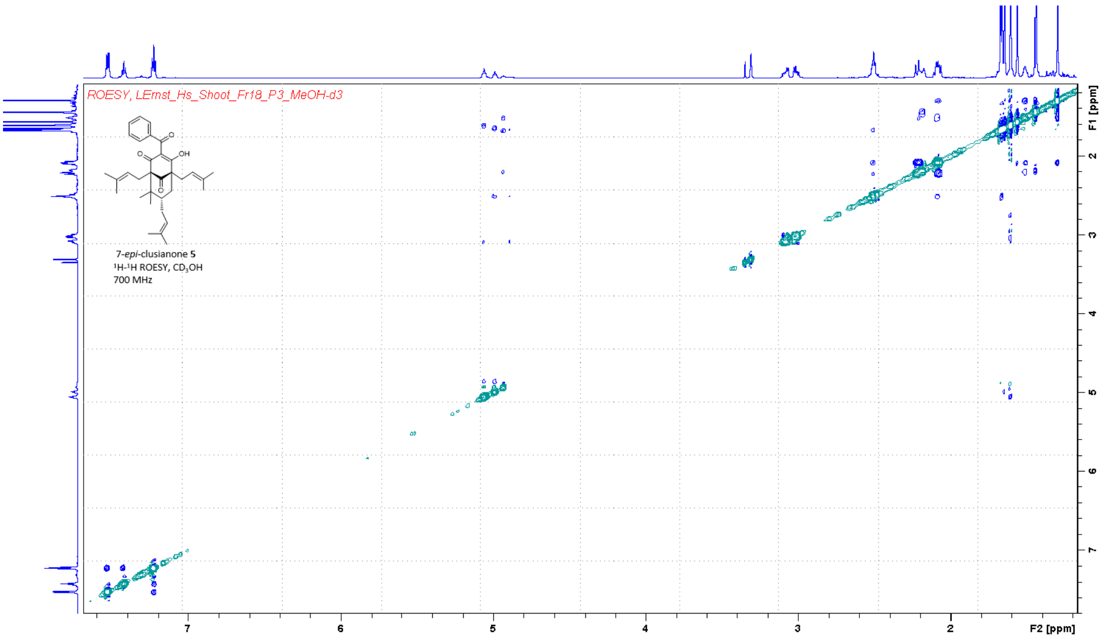
ROESY, LErnst_Hs_Shoot_Fr18_P3_MeOH-d3
7-epi-clusianone 5
1H-1H ROESY, CD3OH
700 MHz
F1 [ppm]
F2 [ppm]

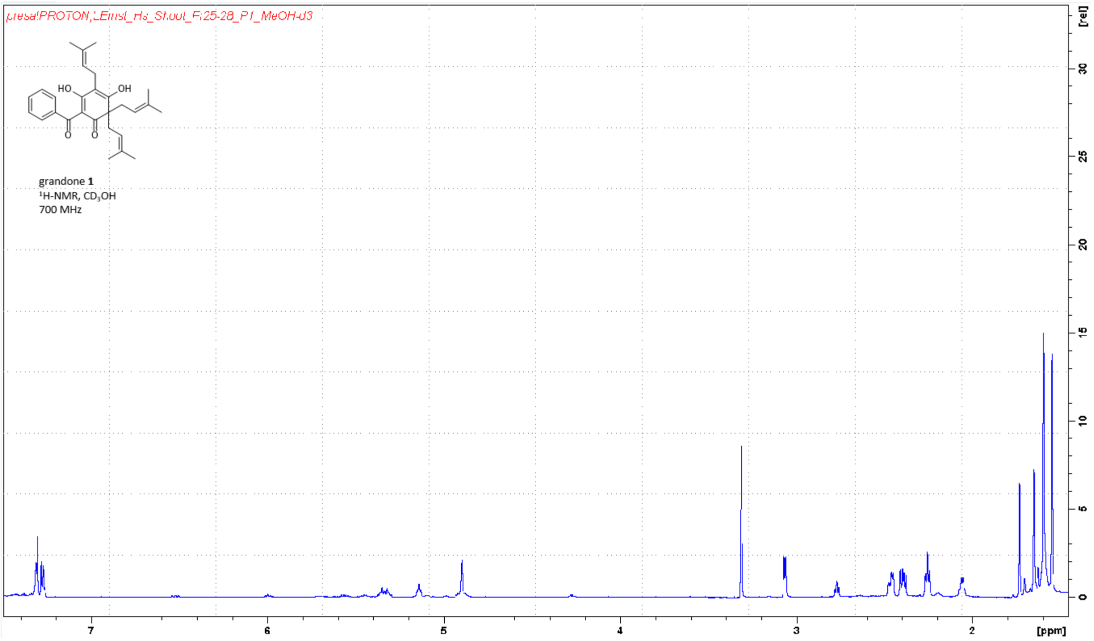
presat PROTON, LErnst_Hs_Shoot_Fr25-28_P1_MeOH-d3
HO
OH
O
O
grandone 1
^{1}H-NMR, CD_3OH
700 MHz
[rel]
[ppm]

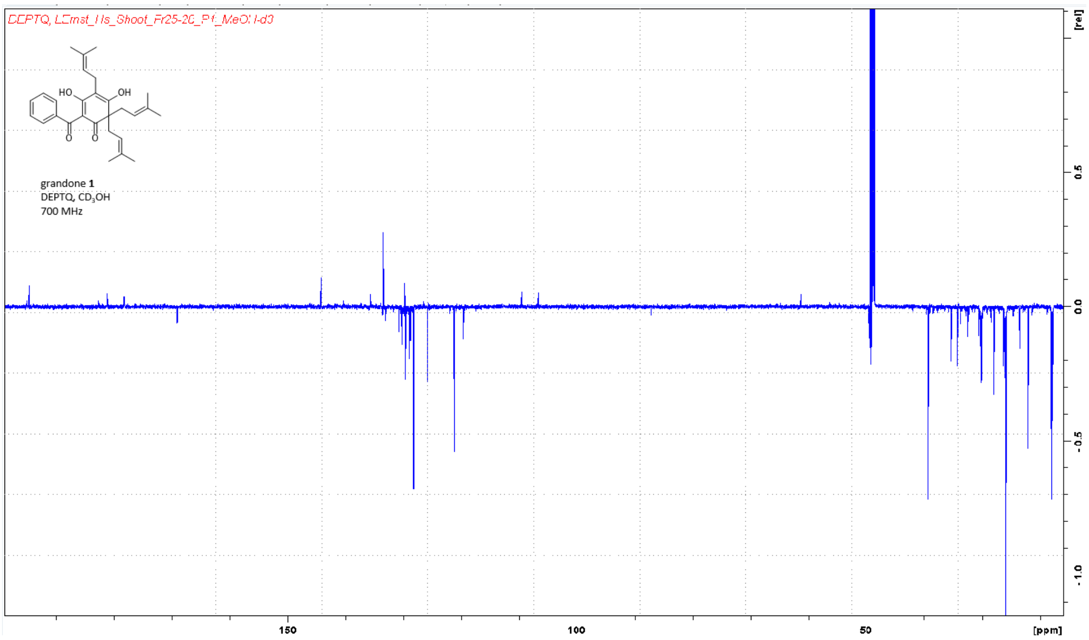
DEPTQ, LErnst_Hs_Shoot_Fr25-28_P1_MeOH-d3
HO
OH
O
O
grandone 1
DEPTQ, CD_3OH
700 MHz
[rel]
[ppm]

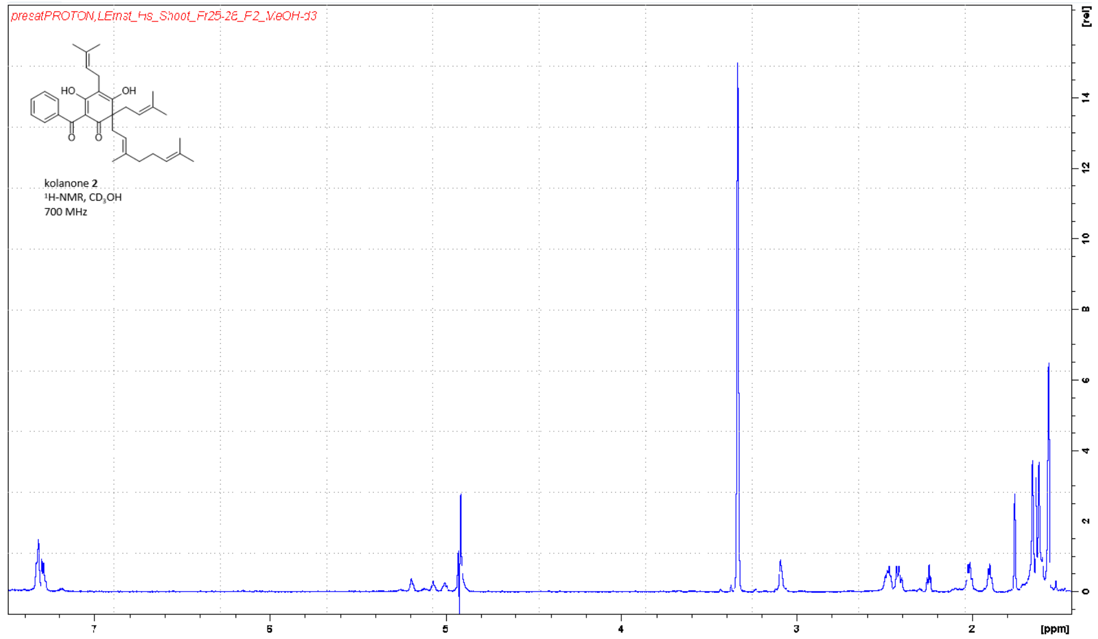
presatPROTON,LErnst_Hs_Shoot_Fr25-28_F2_MeOH-d3
HO
OH
kolanone 2
1H-NMR, CD3OH
700 MHz
[rel]
[ppm]

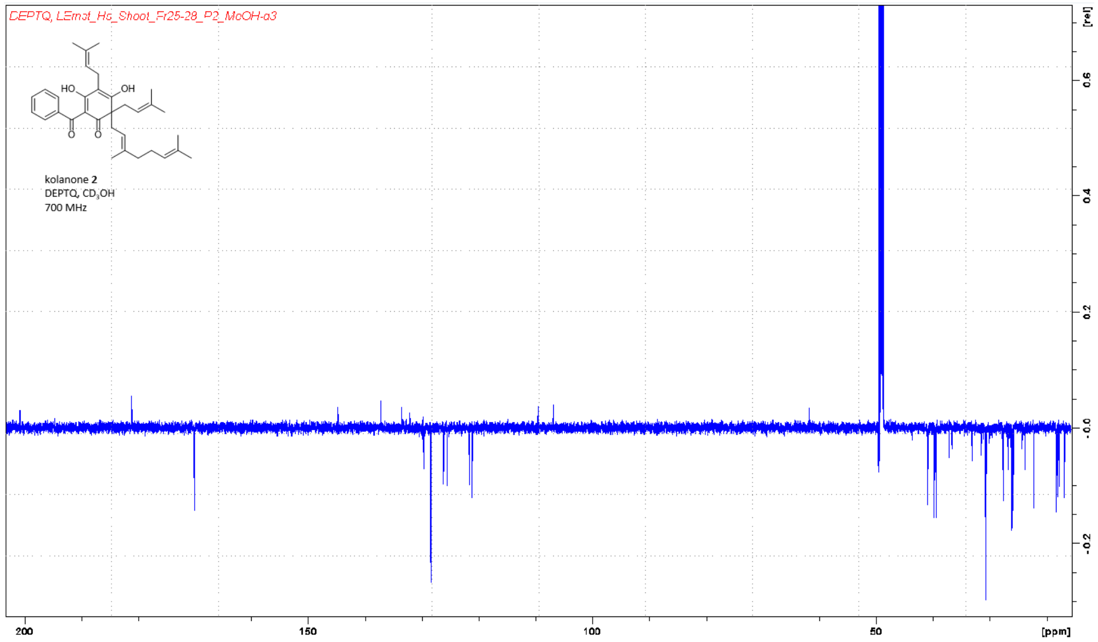
DEPTQ, LErnst_Hs_Shoot_Fr25-28_F2_MeOH-d3
HO
OH
kolanone 2
DEPTQ, CD3OH
700 MHz
[rel]
[ppm]

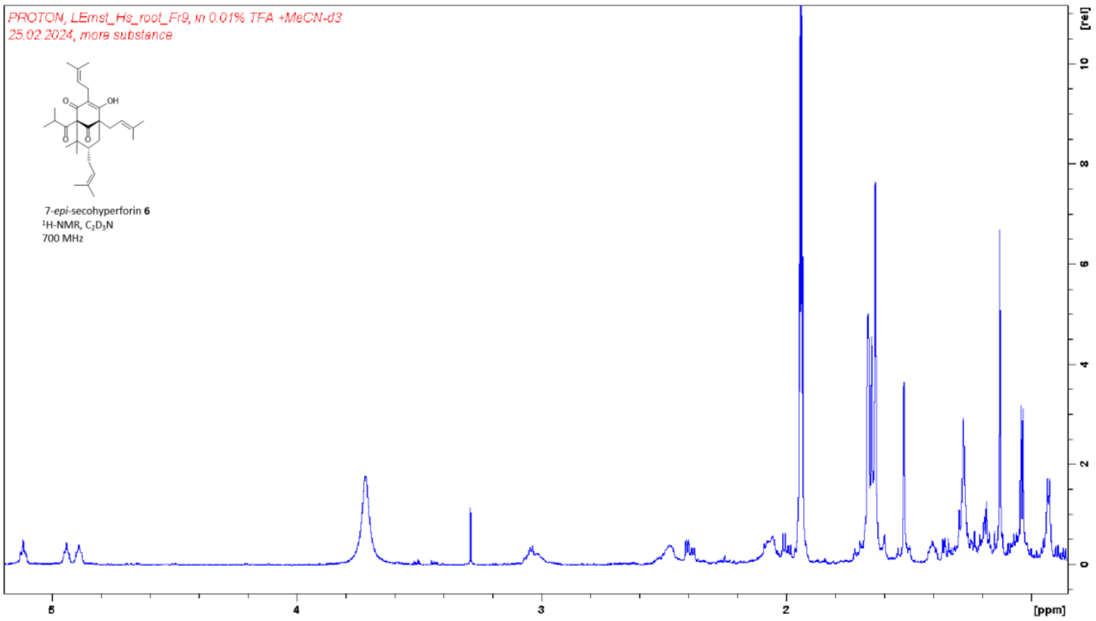
PROTON, LErnst_Hs_root_Fr9, in 0.01% TFA +MeCN-d3
25.02.2024, more substance
7-*epi*-secohyperforin **6**
^{1}H-NMR, C_2D_3N
700 MHz
[rel]
[ppm]

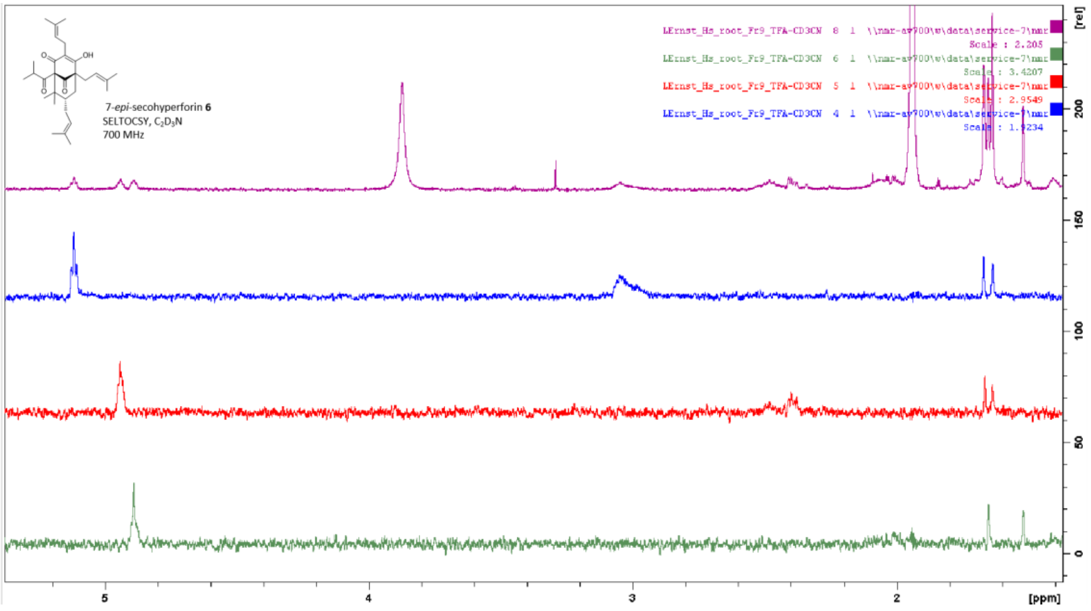
7-*epi*-secohyperforin **6**
SELTOCSY, C_2D_3N
700 MHz
[rel]
[ppm]

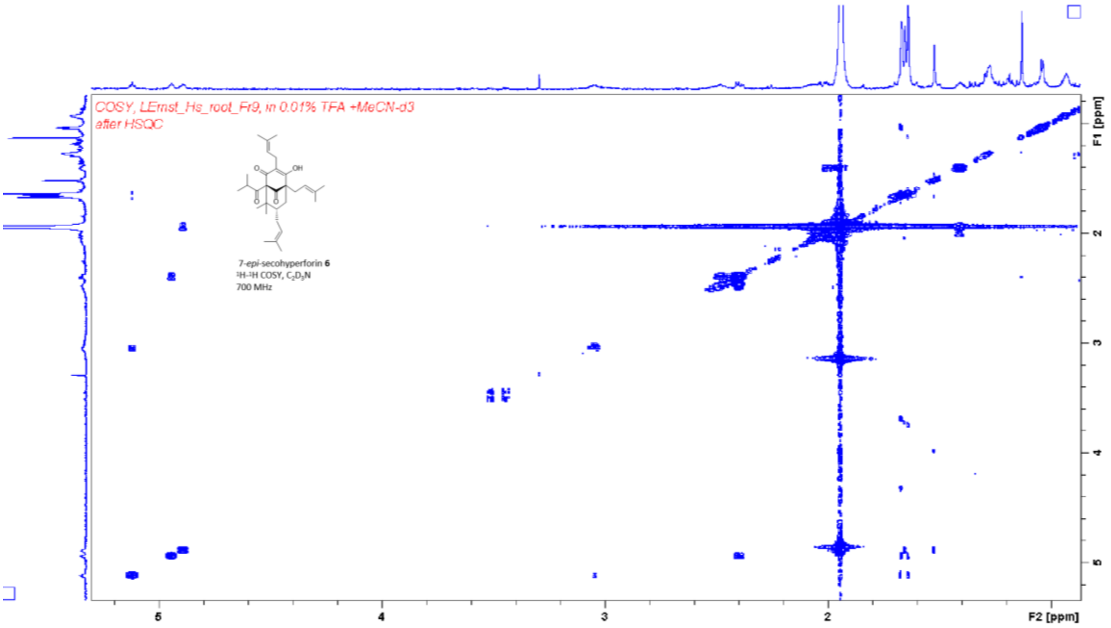
COSY, LErnst_Hs_root_Fr9, in 0.01% TFA +MeCN-d3
after HSQC
7-epi-secohyperforin 6
^{1}H-^{1}H COSY, C_2D_3N
700 MHz
F1 [ppm]
F2 [ppm]

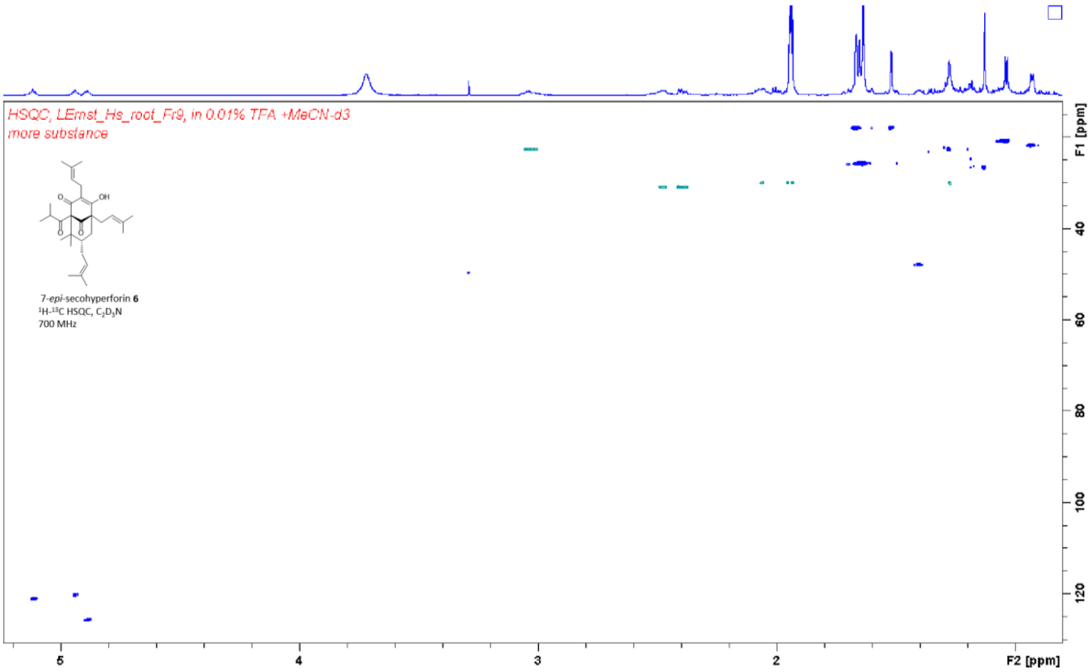
HSQC, LErnst_Hs_root_Fr9, in 0.01% TFA +MeCN-d3
more substance
7-epi-secohyperforin 6
^{1}H-^{13}C HSQC, C_2D_3N
700 MHz
F1 [ppm]
F2 [ppm]

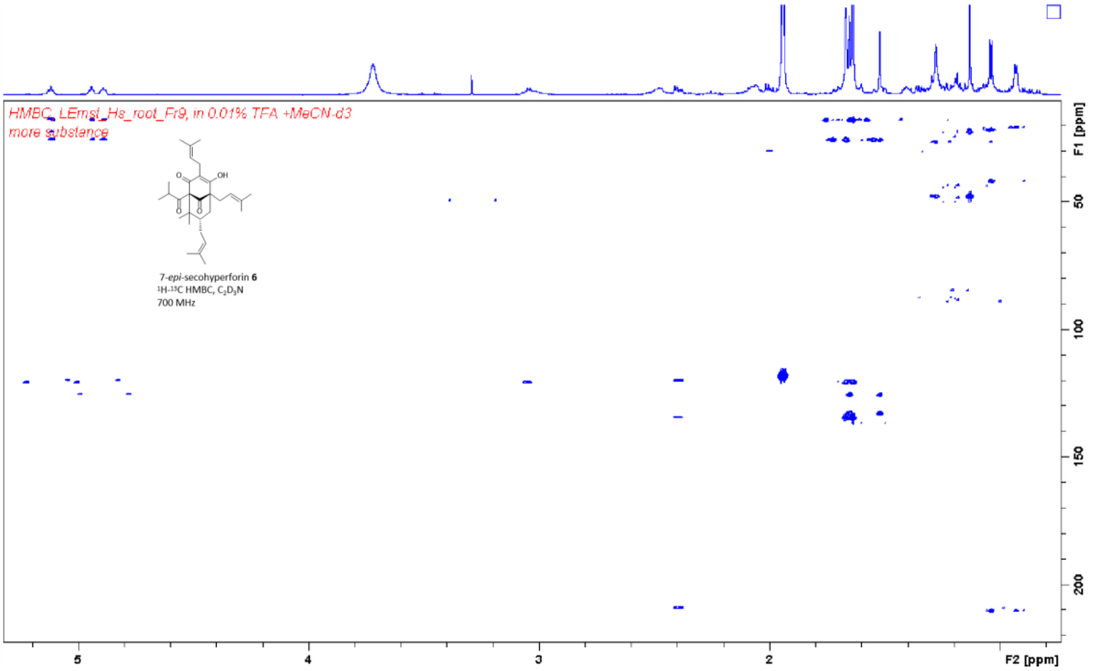

Supplementary References

1. Nagia, M. et al. Sequential regiospecific gem-diprenylation of tetrahydroxyxanthone by prenyltransferases from *Hypericum* sp. *New Phytol.* **222,** 318–334; 10.1111/nph.15611 (2019).

2. Bittrich, V., Amaral, M. d. C. E., Machado, S. M. F. & Marsaioli, A. J. Floral resin of *Tovomitopsis saldanhae* (Guttiferae) and 7-*epi*-nemorosone: structural revision. *Z. Naturforsch. C J. Biosci.* **58,** 643–648; 10.1515/znc-2003-9-1008 (2003).

3. Piccinelli, A. L. et al. Structural revision of clusianone and 7-*epi*-clusianone and anti-HIV activity of polyisoprenylated benzophenones. *Tetrahedron* **61,** 8206–8211; 10.1016/j.tet.2005.06.030 (2005).

4. Tian, W.-J. et al. Hypersampsones S–W, new polycyclic polyprenylated acylphloroglucinols from *Hypericum sampsonii*. *RSC Adv.* **6,** 50887–50894; 10.1039/C5RA26332H (2016).

5. Zeng, Y. H. et al. Geranyl bearing polyisoprenylated benzoylphloroglucinol derivatives from *Hypericum sampsonii*. *Chem. Lett.* **38,** 440–441; 10.1246/cl.2009.440 (2009).

6. Cuesta-Rubio, O., Velez-Castro, H., Frontana-Uribe, B. A. & Cárdenas, J. Nemorosone, the major constituent of floral resins of *Clusia rosea*. *Phytochemistry* **57,** 279–283; 10.1016/S0031-9422(00)00510-0 (2001).

7. Oliveira, C. M. de, Porto, A., Bittrich, V., Vencato, I. & Marsaioli, A. J. Floral resins of clusia spp.: Chemical composition and biological function. *Tetrahedron Lett.* **37,** 6427–6430; 10.1016/0040-4039(96)00656-9 (1996).

8. Lokvam, J., Braddock, J. F., Reichardt, P. B. & Clausen, T. P. Two polyisoprenylated benzophenones from the trunk latex of *Clusia grandiflora* (Clusiaceae). *Phytochemistry* **55,** 29–34; 10.1016/S0031-9422(00)00193-X (2000).

9. Charchoglyan, A. et al. Differential accumulation of hyperforin and secohyperforin in Hypericum perforatum tissue cultures. *Phytochemistry* **68,** 2670–2677; 10.1016/j.phytochem.2007.06.004 (2007).
