# Supplementary material for: Regiodivergent biosynthesis of bridged bicyclononanes
Source: Nat Commun. 2024 May 28;15:4525. doi: 10.1038/s41467-024-48879-w (PMC11133429; doi:10.1038/s41467-024-48879-w)
Supplement: Supplementary file 3 — Reporting Summary [file 41467_2024_48879_MOESM3_ESM.pdf]

nature portfolio

Corresponding author(s): Lukas Ernst, Islam El-Awaad, and Benye Liu

Last updated by author(s): May 4, 2024

Reporting Summary

Nature Portfolio wishes to improve the reproducibility of the work that we publish. This form provides structure for consistency and transparency in reporting. For further information on Nature Portfolio policies, see our Editorial Policies and the Editorial Policy Checklist.

Statistics

For all statistical analyses, confirm that the following items are present in the figure legend, table legend, main text, or Methods section.

n/a	Confirmed	
☐	☒	The exact sample size (*n*) for each experimental group/condition, given as a discrete number and unit of measurement
☐	☒	A statement on whether measurements were taken from distinct samples or whether the same sample was measured repeatedly
☒	☐	The statistical test(s) used AND whether they are one- or two-sided<br>*Only common tests should be described solely by name; describe more complex techniques in the Methods section.*
☒	☐	A description of all covariates tested
☒	☐	A description of any assumptions or corrections, such as tests of normality and adjustment for multiple comparisons
☐	☒	A full description of the statistical parameters including central tendency (e.g. means) or other basic estimates (e.g. regression coefficient) AND variation (e.g. standard deviation) or associated estimates of uncertainty (e.g. confidence intervals)
☒	☐	For null hypothesis testing, the test statistic (e.g. *F*, *t*, *r*) with confidence intervals, effect sizes, degrees of freedom and *P* value noted<br>*Give P values as exact values whenever suitable.*
☒	☐	For Bayesian analysis, information on the choice of priors and Markov chain Monte Carlo settings
☒	☐	For hierarchical and complex designs, identification of the appropriate level for tests and full reporting of outcomes
☒	☐	Estimates of effect sizes (e.g. Cohen's *d*, Pearson's *r*), indicating how they were calculated

Our web collection on statistics for biologists contains articles on many of the points above.

Software and code

Policy information about availability of computer code

Data collection	All data were collected using commercially available software. HPLC-DAD data were acquired by EZChrom Elite 3.3.2 SP1 (Agilent, Santa Clara, USA). Mass spectrometry data were collected using Analyst v1.64.2 (Applied Biosystems/MDS SCIEX, Darmstadt, Germany) or Compass HyStar (Bruker Daltonics, Bremen, Germany). RT-qPCR data were acquired using Bio-Rad CFX Manager v3.1 (Bio-Rad). Confocal laserscanning microscopy images were recorded by ZEN black edition 2.1 (Zeiss, Oberkochen, Germany). NMR data was aquired using Bruker TopSpin v3.6.1.
Data analysis	All data analysis was conducted using either commercial or previously published software. Work-up and assembly of RNA-seq data was performed using Trimmomatic v0.38.1, FastQC v0.74 and Trinity v2.9.1. Transcript abundance was determined using Salmon v1.5.1. Kinetics and RT-qPCR data were analysed using OriginPro2022 (OriginLab, Northampton, USA). NMR spectra were interpreted using Bruker TopSpin v3.6.1. Phylogenetic analysis was performed using MEGA11 v11.0.13. Homology models were obtained using AlphaFold2 (DeepMind, London, England), refined using Prime (Schrödinger, New York, USA) and validated using the SAVES6 protein check server (https://saves.mbi.ucla.edu/). Ligand preparation and docking simulations were carried out using Schrödinger's suite of molecular modelling tools (Schrödinger Release 2018-2).

For manuscripts utilizing custom algorithms or software that are central to the research but not yet described in published literature, software must be made available to editors and reviewers. We strongly encourage code deposition in a community repository (e.g. GitHub). See the Nature Portfolio guidelines for submitting code & software for further information.

Data

Policy information about availability of data

All manuscripts must include a data availability statement. This statement should provide the following information, where applicable:

- Accession codes, unique identifiers, or web links for publicly available datasets
- A description of any restrictions on data availability
- For clinical datasets or third party data, please ensure that the statement adheres to our policy

All reported data within this study were uploaded to publicly available databases or are available upon request. The coding sequences of genes characterized in this study are deposited in the National Center for Biotechnology Information (NCBI) GeneBank database under the accession numbers OR464113 (HsCPTa), OR464114 (HsCPTb), and PP538028 (HpCPTa1). The raw reads obtained from RNA sequencing of H. sampsonii root and shoot samples are deposited in the NCBI Sequence Read Archive under the BioProject accession PRJNA1010338.

Research involving human participants, their data, or biological material

Policy information about studies with human participants or human data. See also policy information about sex, gender (identity/presentation), and sexual orientation and race, ethnicity and racism.

Reporting on sex and gender	No human research participants are involved in this study.
Reporting on race, ethnicity, or other socially relevant groupings	No human research participants are involved in this study.
Population characteristics	No human research participants are involved in this study.
Recruitment	No human research participants are involved in this study.
Ethics oversight	No human research participants are involved in this study.

Note that full information on the approval of the study protocol must also be provided in the manuscript.

Field-specific reporting

Please select the one below that is the best fit for your research. If you are not sure, read the appropriate sections before making your selection.

☒ Life sciences ☐ Behavioural & social sciences ☐ Ecological, evolutionary & environmental sciences

For a reference copy of the document with all sections, see nature.com/documents/nr-reporting-summary-flat.pdf

Life sciences study design

All studies must disclose on these points even when the disclosure is negative.

Sample size	No statistical methods were used to predetermine sample size. Enzyme kinetics data were obtained from three independent experiments to ensure that each data point was reproducible. For quantification of expression and metabolite levels, three experiments were performed with material from two different plants (n=6).
Data exclusions	No data were excluded from the analysis of experiments that assess averages of data points. Some figures, such as chromatograms, MS/MS fragmentations or UV spectra show exemplary examples of a given reproducible result.
Replication	Replicates of three were chosen for most experiments presented in this study, including enzyme characterizations, quantification of enzyme products, and heterologous expression in N. benthamiana. For the purpose of mutant activity screening , product yields of some reciprocal mutants are given as obtained from single standardized assays, however, their activity was confirmed at least in two reactions done on different days.
Randomization	Randomization was not relevant for this study, as no experiment required randomized allocation to experimental groups. Plants used for heterologous expression studies were grown at equal conditions and the two youngest fully expanded leaves from each plant were used for experiments.
Blinding	Blinding was not relevant for this study, as the insight of the investigators was required to functionally characterize gene products or structurally elucidate metabolites.

Reporting for specific materials, systems and methods

We require information from authors about some types of materials, experimental systems and methods used in many studies. Here, indicate whether each material, system or method listed is relevant to your study. If you are not sure if a list item applies to your research, read the appropriate section before selecting a response.

Materials & experimental systems

n/a	Involved in the study
☒	☐ Antibodies
☒	☐ Eukaryotic cell lines
☒	☐ Palaeontology and archaeology
☒	☐ Animals and other organisms
☒	☐ Clinical data
☒	☐ Dual use research of concern
☐	☒ Plants

Methods

n/a	Involved in the study
☒	☐ ChIP-seq
☒	☐ Flow cytometry
☒	☐ MRI-based neuroimaging
